# Cysteine: an overlooked energy and carbon source

**DOI:** 10.1038/s41598-021-81103-z

**Published:** 2021-01-25

**Authors:** Luise Göbbels, Anja Poehlein, Albert Dumnitch, Richard Egelkamp, Cathrin Kröger, Johanna Haerdter, Thomas Hackl, Artur Feld, Horst Weller, Rolf Daniel, Wolfgang R. Streit, Marie Charlotte Schoelmerich

**Affiliations:** 1 Microbiology and Biotechnology, Institute of Plant Sciences and Microbiology, University of Hamburg, 22609 Hamburg, Germany; 2 Genomic and Applied Microbiology and Göttingen Genomics Laboratory, Georg-August University Göttingen, Grisebachstraße 8, 37077 Göttingen, Germany; 3 Institute of Organic Chemistry, University of Hamburg, Martin-Luther-King-Platz 6, 20146 Hamburg, Germany; 4 Institute of Physical Chemistry, University of Hamburg, Grindelallee 117, 20146 Hamburg, Germany

**Keywords:** Microbiology, Applied microbiology, Bacteria, Environmental microbiology, Nanobiotechnology, Nanoparticles, Biotechnology, Biomineralization

## Abstract

Biohybrids composed of microorganisms and nanoparticles have emerged as potential systems for bioenergy and high-value compound production from CO_2_ and light energy, yet the cellular and metabolic processes within the biological component of this system are still elusive. Here we dissect the biohybrid composed of the anaerobic acetogenic bacterium *Moorella thermoacetica* and cadmium sulphide nanoparticles (CdS) in terms of physiology, metabolism, enzymatics and transcriptomic profiling. Our analyses show that while the organism does not grow on l-cysteine, it is metabolized to acetate in the biohybrid system and this metabolism is independent of CdS or light. CdS cells have higher metabolic activity, despite an inhibitory effect of Cd^2+^ on key enzymes, because of an intracellular storage compound linked to arginine metabolism. We identify different routes how cysteine and its oxidized form can be innately metabolized by the model acetogen and what intracellular mechanisms are triggered by cysteine, cadmium or blue light.

## Introduction

Anaerobic microorganisms play a pivotal role in the global cycling of elements and nutrients, are key in determining the health of animals and humans and are important players for biotechnological and industrial processes to solve tomorrow’s challenges of pollution, resource scarcity and energy requirements^1,2^. An innovative proof-of-concept study has recently been described in which a non-photosynthetic anaerobic bacterium becomes photosynthetic by coupling it to CdS nanoparticles^3^. This poses a very appealing technology, as the model organism used is an acetogen and these organisms have emerged as microbial platforms for the production of high-value compounds (biofuel, bioplastic) from CO_2_ and inexpensive energy sources^4^. Just like methanogens and sulfate reducers, acetogens use the primordial Wood–Ljungdahl pathway for CO_2_ fixation. In the biohybrid system, the model acetogen *Moorella thermoacetica* apparently uses light energy to drive CO_2_ fixation when covered in microbially-formed CdS nanoparticles^3^. The electrons (e^−^) are described to originate from illuminated CdS and the leftover hole pair is then quenched by the sacrificial reducing agent cysteine, leading to the oxidized disulfide form, cystine (CySS). A scheme of the proposed biohybrid system is depicted in Supplementary Fig. 1. The elegant combination of chemistry and biology and promising potential application of this technology sparked us to investigate it.

Acetogens rely on the WLP to fix two moles of CO_2_ to one mole of acetyl-CoA using eight electrons. In the methyl-branch, CO_2_ is reduced to formate (2e^−^), which is activated to formyl-THF at the cost of ATP, and further converted via methenyl-THF and methylene-THF (2e^−^) to methyl-THF (2e^−^). In the carbonyl-branch, another molecule of CO_2_ is reduced (2e^−^) to enzyme-bound CO and both C_1_ moieties are then unified by the bifunctional carbon monoxide dehydrogenase/acetyl-CoA synthase (CODH/Acs) to acetyl-CoA. The conversion of acetyl-CoA via acetyl-phosphate to acetate finally yields ATP from substrate-level phosphorylation (SLP). The electrons are derived from inorganic molecular hydrogen (H_2_) or carbon monoxide (CO) when acetogens grow chemolithoautotrophically. They can also utilize a spectrum of organic compounds ranging from sugars, organic acids, alcohols to methoxylated compounds to derive additional electrons for CO_2_ fixation and more acetyl-CoA precursors for acetate formation^5^. Since the WLP is overall energy-neutral, the chemiosmotic mechanism is the only source of ATP synthesis when acetogens sustain a chemolithoautotrophic lifestyle^6^. Their respiratory chain is very simple, often comprising only a redox-driven ion pump in conjunction with an ion-gradient-depleting ATP synthase. In the mesophilic acetogen *Acetobacterium woodii*, this ion pump is the Rnf complex, which couples the exergonic electron flow from reduced ferredoxin (Fd^2−^) onto NAD^+^ to the establishment of an electrochemical Na^+^ gradient ($\tilde \upmu$Na^+^) and this $\tilde \upmu$Na^+^ gradient fuels a Na^+^-dependent F_1_F_O_ ATP synthase^7^. The thermophilic acetogen *Thermoanaerobacter kivui* on the other hand uses an Ech complex, which couples the electron flow of Fd^2−^ onto H^+^ to the establishment of an ion gradient composed of both Na^+^ and H^+^, and it is only the latter which fuels a H^+^-dependent F_1_F_O_ ATP synthase^8^. Just like *T. kivui, M. thermoacetica* also encodes an Ech-ATPase circuit, but additionally possesses quinones and cytochromes that may be part of the respiratory chain as well^9,10^.

In this paper we examine *M. thermoacetica* in context of the biohybrid system by assessing the influence of cadmium, light and cysteine on microbial growth, metabolic activity, enzyme activity and the transcriptome of *M. thermoacetica*.

## Results

### Impact of cadmium, light and cysteine on growth

First experiments were set out to assess growth of *M. thermoacetica* in dependence of all factors within the biohybrid system: cadmium, blue light and l-cysteine. All cells were grown in complex medium with 50 mM d-glucose. Exposure of growing cells to blue light (440 nm peak, 78 × 10^17^ photons m^−2^ s^−1^, as in^3^) or cadmium (1 mM CdCl_2_) did not show a significant effect on growth (doubling times [t_d_] of 7.2 and 6.9 or 6.4 and 5.9 and final optical densities (OD_600_) of 0.68 or 0.63 or a cell count of 5.9 or 6.0 × 10^5^ for cells grown with and without light or with and without cadmium) (Supplementary Fig. 2A). Additional cysteine (20 mM l-cysteine) did not significantly affect growth, indicating that cysteine is not growth-supportive (t_d_ of 8.0 or 8.9 h; final OD_600_ of 1.08 or 0.97) (Supplementary Fig. 2B). Cultures also did not grow on l- or d-cysteine alone (when inoculated from glucose or glucose + H_2_ + CO_2_ pregrown cultures) and mixotrophic conditions with substrate-limiting concentrations of d-glucose (10 mM) and l-cysteine as additional growth substrate did not result in an increased growth either (Supplementary Fig. 2C).

**Figure 1:**
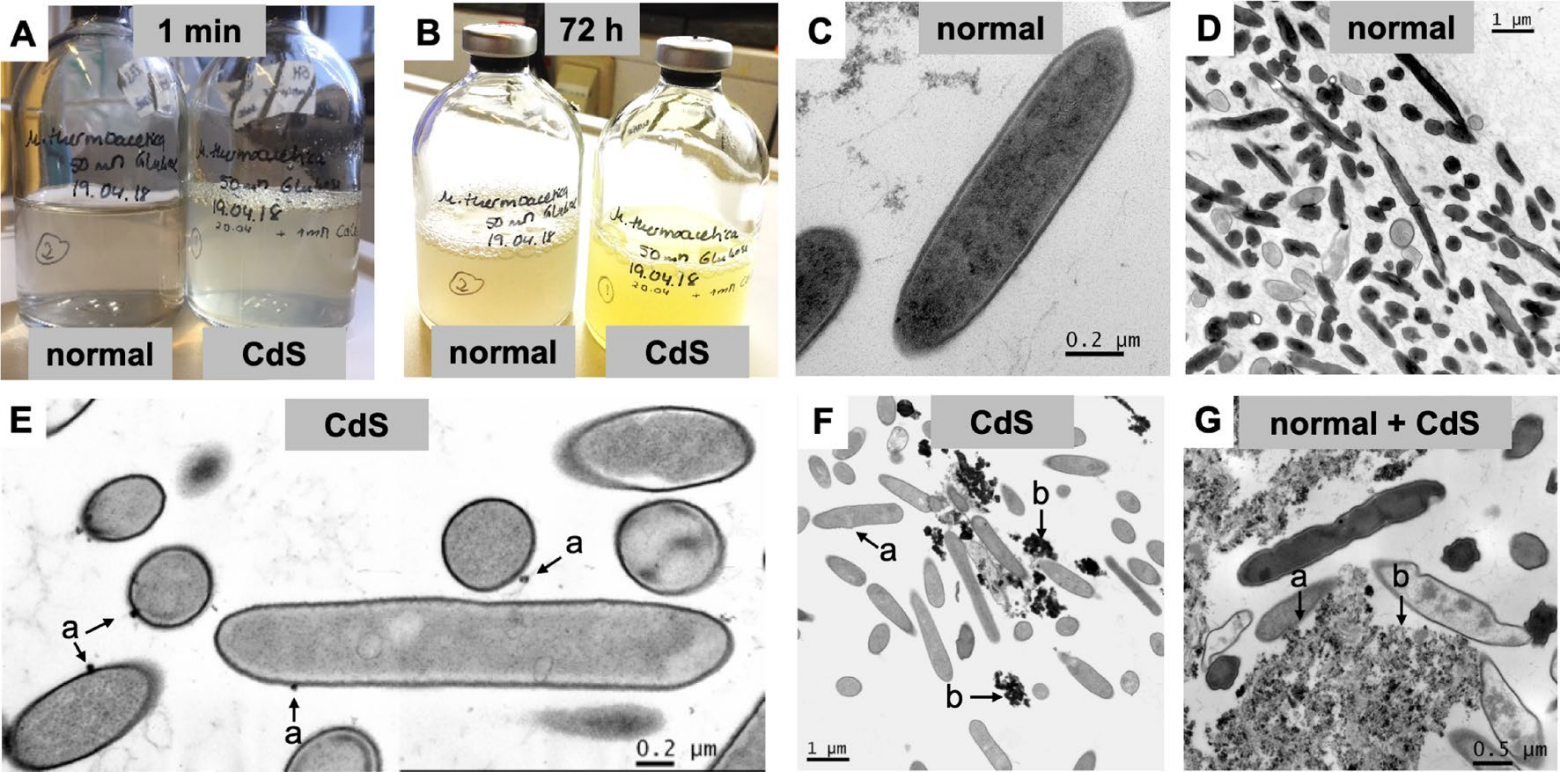
Normal, CdS and normal + CdS cells of *M. thermoacetica*. *M. thermoacetica* was cultivated in complex medium and supplemented with CdCl_2_ to induce CdS nanoparticle formation. A, B. Image of cells growing without (left) or with (right) CdCl_2_ 1 min (**A**) or 72 h (**B**) after supplementation. (**C**–**G**) TEM images of normal, CdS cells or normal cells supplemented with chemically synthesized CdS (normal + CdS) from AAP1 at the end of the experiment. Arrows highlight nanoparticles attached to the surface of the cells (a) or extracellular cadmium precipitate (b).

Cadmium-supplemented cultures of *M. thermoacetica* and *T. kivui* turned dark yellow, as characteristic for CdS^11^ (Fig. 1A,B, Supplementary Fig. 4A). The nanoparticle formation is a consequence of the enzymatic liberation of sulfide from cysteine by a cysteine desulfhydrase in the organism, which precipitates with the Cd^2+^. This is an ATP-dependent process, since only growing cells form CdS^11^. Transmission electron microscopy (TEM) images of growing *M. thermoacetica* showed that nanoparticle formation commenced 6 h after cadmium supplementation, CdS nanoparticles accumulated outside of the cells and growing cells looked comparable with or without cadmium (Supplementary Fig. 3). The CdS nanoparticles were partly attached to the surface of the cells (Fig. 1E) or as extracellular cadmium precipitate (Fig. 1F). When normal cells were incubated with chemically synthesized CdS (normal + CdS), large extracellular precipitates were observed as well, with cells in close proximity (Fig. 1G).

### Dissection of metabolic activity in the CdS-*M. thermoacetica* biohybrid system

**Figure 2:**
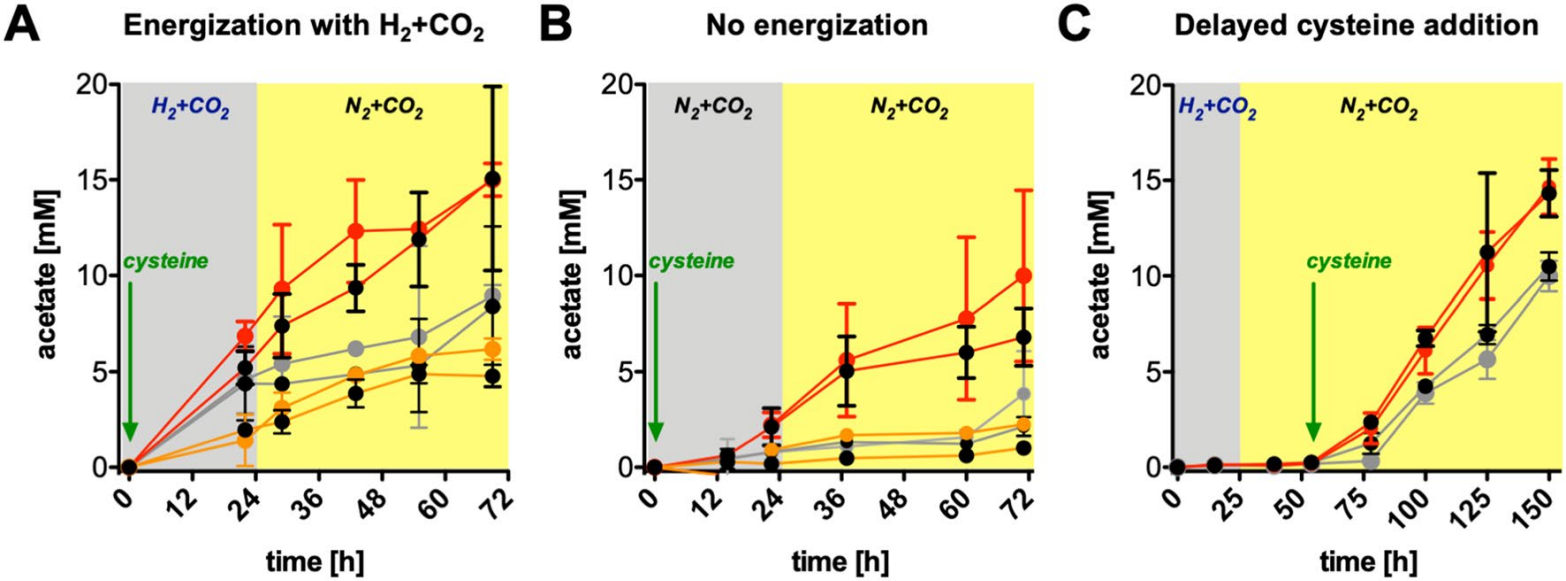
Metabolic activity of CdS cells, normal cells or normal + CdS cells under photosynthetic conditions. *M. thermoacetica* was grown on 50 mM d-glucose with or without 1 mM CdCl_2_ supplementation after 24 h. Cells were harvested after 72 h and transferred into photosynthetic medium at 1 mg/ml. Red lines depict CdS cells, grey lines depict normal cells and orange lines depict normal cells supplemented with chemically synthesized nanoparticles in the beginning of the experiment (at 1 or 0.5 mM CdS in **A** or **B**). The assays were incubated at 55 °C in a shaking water bath in blue light (coloured circles) or in the dark (black circles) in a 1.3 × 10^5^ Pa H_2_ + CO_2_ (**A**,**C**) or N_2_ + CO_2_ (80:20 [v/v]) (**B**) atmosphere. All assays were supplemented with 6 mM l-cysteine as indicated by the arrows. N = 2 (**A**,**B**) or 3 (**C**), SD.

CdS-*M. thermoacetica* (biohybrid or CdS) cells or normal *M. thermoacetica* cells (control) were harvested in the early stationary phase (to facilitate sufficient ripening of the nanoparticles) and transferred into photosynthetic medium (as described previously^3^). Three sets of acetogenic artificial photosynthesis (AAP) experiments were performed following the experimental setup described in Sakimoto et al*.* (2016), to dissect the metabolic activity of CdS or normal cells in dependence of light, l-cysteine and energization with H_2_ + CO_2_. Moreover, assays were also performed with normal cells which were incubated with chemically synthesized CdS nanoparticles (Supplementary Fig. 4). In a first AAP experiment (AAP1), CdS cells, normal cells or normal + CdS cells were supplemented with l-cysteine and incubated in the darkness with H_2_ + CO_2_ for 24 h, then the gas phase was exchanged to N_2_ + CO_2_ and one set of cells was further incubated under blue light, while a control set remained in the dark. CdS cells of *M. thermoacetica* looked more intact at the end of the experiments than normal or normal + CdS cells (Fig. 1C–G). This was also true for CdS cells of *T. kivui* compared to normal cells (Supplementary Fig. 5). All assays containing CdS (biologically or chemically formed) revealed that they were not within the cells and mostly aggregated extracellularly. Acetate was produced from the beginning of the incubation in all assays (Fig. 2A). Acetate formation was the same (rate and yield) in CdS cells incubated with or without light (Table 1). Normal cells also produced acetate, but at lower levels (48–55%) than CdS cells, and both rate and final acetate concentration were 1.1-fold stimulated by light. Normal + CdS cells produced less acetate than the normal cells (57–70%) at slightly decreased rates and both rate and final acetate concentration were 1.3-fold stimulated by light (Fig. 2A). The observation that normal cells performed comparably with or without chemically synthesized nanoparticles suggests that the increased metabolic activity of CdS cells was due to an intracellular storage compound fuelling metabolism. This compound must have been produced from glucose during growth in response to cadmium exposure. This would also explain why CdS cells looked more intact at the end of the AAP experiments than normal cells (with or without chemically synthesized CdS), since the stored compound served as an additional energy source.

**Table 1:** Acetate production in the AAP assays. N = 2 (A, B*) or 3 (C); SD. *normal + CdS assays in B were only performed as one biological assay.

Acetate production	A: Energization with H_2_ + CO_2_		B: No energization		C: Delayed cysteine addition	
	[µM/h]	[mM]	[µM/h]	[mM]	[µM/h]	[mM]
CdS_light_	213 ± 28	15.0 ± 0.9	152 ± 27	10.0 ± 4.5	157 ± 15	14.7 ± 2.5
CdS_dark_	213 ± 24	15.1 ± 4.8	103 ± 13	6.8 ± 1.5	155 ± 14	14.3 ± 1.2
Normal_light_	117 ± 25	8.9 ± 0.6	45 ± 11	3.8 ± 2.2	105 ± 9	10.0 ± 0.8
Normal_dark_	103 ± 24	8.4 ± 4.2	26 ± 4	2.1 ± 0.5	110 ± 6	10.5 ± 0.7
Normal + CdS_light_	99 ± 10	6.2 ± 0.6	33 ± 6	2.3*	–	–
Normal + CdS_dark_	75 ± 7	4.8 ± 0.6	13 ± 2	1.0*	–	–

Since our experiments did not consolidate that the biohybrid system makes acetate from light energy, we carefully compared our experimental conditions and results to the initial report^3^. The experimental conditions were identical with the following variations: 55 instead of 52 °C, DSM 521 instead of ATCC39073 strain and a normalized protein content in every assay (1 mg/ml) instead of the same volumes of culture (10 ml culture). The most important difference regarding our results was that the initial report describes that only CdS hybrid cells exposed to light produced acetate, yielding a final Δacetate concentration of ~ 1.3 mM after 3 days. Unfortunately, it is not clear whether the Δ concentration is a result of subtracting the (a) initial acetate concentration after H_2_ + CO_2_ energization or (b) acetate produced by normal cells incubated with light at each respective time point (this would explain the same error bars for each time point). In our experiments, the level of acetate production was much higher (11- or 5-fold without or with subtracting acetate produced by normal cells), which may be because more cells were present in our assays. Most importantly, we saw acetate formation also by the normal cells, and CdS cells kept in the dark equally produced acetate, while these depleted acetic acid in the initial report.

Due to our different results, we decided to carefully assess the biohybrid system more closely. In a second AAP experiment, AAP1 was repeated, but without the initial energization phase with H_2_ + CO_2_, since this is an additional energy/electron source for the organism. Again, acetate was produced from the beginning of the incubation in all assays and lower in normal cells than CdS cells (Fig. 2B). Light stimulated the rate and yield of acetate production 1.5 and 1.7-fold in CdS and normal cells (and even 2.3 and 2.5-fold in normal + CdS cells, but this last assay was only performed once) (Table 1). Therefore, this experiment verified the observed effects in AAP1 and additionally demonstrated that H_2_ is an electron source for metabolism in the system and accountable for the increased acetate formation in AAP1 compared to AAP2. The data suggest that the stimulation of metabolism by light may be primarily a consequence of stimulated enzyme activity. The third AAP experiment was a repetition of AAP1, with the exception that cysteine was not present from the beginning, but only supplemented after 52 h (Fig. 2C). This time, acetate formation only started at the time when cysteine was added (52 h). Furthermore, light had no significant effect on the rate and final acetate formed by CdS or normal cells (Table 1, Fig. 2C). Therefore, the AAP3 experiment consolidated a stimulation of metabolism by CdS (but not light) and revealed that acetate formation was strictly dependent on l-cysteine.

The same trends were observed for experiments performed with biohybrid and normal cells of *T. kivui* (Supplementary Fig. 6). Acetate formation was independent of light, highly stimulated (rate and yield) with H_2_ as additional energy source, and higher in assays containing CdS cells than normal cells. Overall, the AAP experiments clearly demonstrated that in our system (i) microbial metabolism was strictly dependent on l-cysteine (ii) CdS cells showed increased metabolic activity, (iii) light could stimulate metabolism, but this was not dependent on CdS and (iv) H_2_ + CO_2_ boosted metabolic activity.

### Key enzyme activities in crude extracts

To assess whether the higher metabolic activity in CdS cells may be a consequence of increased enzyme activities, we performed assays using crude extracts (CE) of *M. thermoacetica.* Since the growth phase can significantly affect enzyme activities (and AAP experiments were performed with cells harvested in the early stationary growth phase herein and in studies by Sakimoto et al*.* (2016)), CEs were prepared from cultures grown with (normal) or without cadmium (CdS) which were harvested in the exponential or stationary growth phase. Subsequently, key enzyme activities were assayed to reflect enzymatic processes central in acetogens. The pyruvate:ferredoxin oxidoreductase (PFOR), which links glycolysis to the WLP (Fig. 5, no. 9); the formate dehydrogenase (FDH), which catalyses the first step of the WLP by reducing CO_2_ to formate (Fig. 5, no. 2); hydrogenase, which is key to balancing reducing equivalents and deriving electrons from H_2_ oxidation and the cystine reductase, which can replenish (chemically oxidized) cysteine (Fig. 5, no. 0).

PFOR (pyruvate:methylene blue) and hydrogenase (H_2_:MV) activity was significantly lower in CEs prepared from CdS cells (CE_CdS_) in the exponential or stationary growth phase (43 or 32% and 29 or 58%), while FDH (formate:NADP^+^) was either lower or higher (83 or 136%) and cystine reductase (H_2_:MV→MV^2−^:cystine) was not affected (103 or 100%) (Table 2). We also tested for alanine dehydrogenase activity as described in^12^ and cysteine desulfhydrase (Csh) activity as described in^13^, but were unable to detect these activities under the conditions tested. Cysteine desulfhydrase activity has been detected in CEs of *M. thermoacetica* grown on glucose previously^11^, but since this assay involves highly toxic components such as 10 mM HgCl_2_, these measurements were not repeated in our study. The lower enzyme activity in CdS cells could be explained by a lower gene expression in response to cadmium, or traces of intracellular Cd^2+^ preventing proper maturation or catalyses. Moreover, the activities were significantly lower in the stationary growth phase than the exponential growth phase for the FDH and hydrogenase, but not the PFOR or cystine reductase. Hence, the connection between the WLP and glycolysis (PFOR) and reduction of cystine to metabolisable cysteine (cystine reductase) was retained when cellular fitness of the population seized, whereas the lower redox state led to decreased enzyme activities involved in discarding electrons (FDH and hydrogenase).

**Table 2:** Specific enzyme activities measured in crude extracts of *M. thermoacetica*. Cells were grown on 50 mM d-glucose with (CdS) or without (normal) 1 mM CdCl_2_ and harvested after 2 (exponential) or 3 (stationary) days of growth. N = 3; SD.

Enzyme	Specific activity [U/mg]			
	Normal		CdS	
	Exponential	Stationary	Exponential	Stationary
PFOR (pyruvate:MB oxidoreductase)	2.88 ± 0.21	3.55 ± 0.07	1.25 ± 0.23	1.12 ± 0.06
FDH (formate:NADP^+^)	0.29 ± 0.03	0.14 ± 0.02	0.24 ± 0.03	0.19 ± 0.03
Hydrogenase (H_2_:MV)	3.21 ± 0.92	1.23 ± 0.11	0.92 ± 0.20	0.71 ± 0.03
Cystine reductase (H_2_:MV→MV^2−^:cystine)	2.88 ± 0.10	2.84 ± 0.02	2.97 ± 0.11	2.85 ± 0.05

### Cysteine metabolisation in resting cells

**Figure 3:**
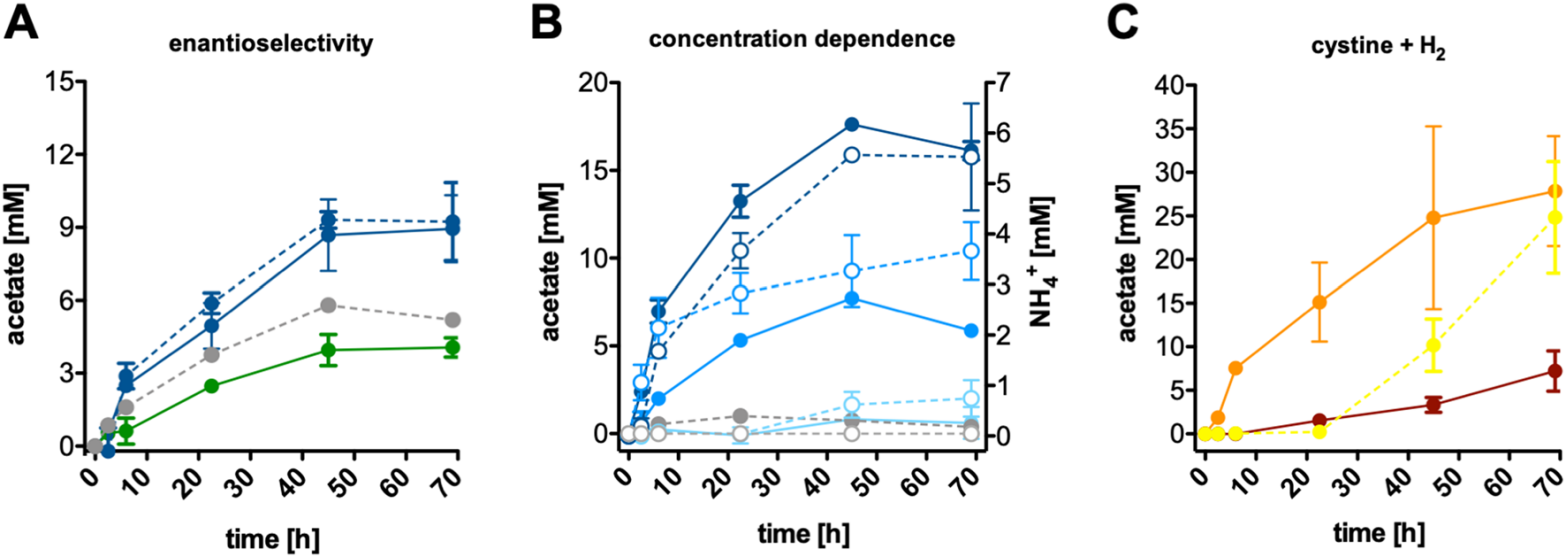
Metabolic activity by resting cells in dependence of cysteine. *M. thermoacetica* was grown on 50 mM d-glucose until the mid-exponential growth phase, cells were harvested and transferred into bicarbonate-containing cell suspension buffer at a final concentration of 1 mg/ml under a 1.3 × 10^5^ Pa N_2_ + CO_2_ (80:20 [v/v]) atmosphere and incubated in the dark at 55 °C under constant shaking (100 rpm). (**A**) Assays were supplemented with 6 mM l-cysteine (blue) or 6 mM d-cysteine (green). A control received no cysteine (grey) and CO_2_-free control assay received 6 mM l-cysteine without bicarbonate in the buffer and a 100% N_2_ gas phase (blue dashed line). (**B**) Assays were supplemented with 3 mM (light blue), 12 mM (blue) or 24 mM (dark blue) l-cysteine. One control assay contained no cells but 3 mM l-cysteine (grey), another contained no cysteine and these values were subtracted from all assays (for correction purposes due to acetate formation from storage compounds). Acetate (solid line) and NH_4_^+^ formation (dashed line) was monitored; (**C**) Assays received 6 mM cystine (red) or 1.3 × 10^5^ Pa H_2_ + CO_2_ (80:20 [v/v]) (yellow) or a combination of both (orange).

Since the AAP experiments suggested that *M. thermoacetica* has an intrinsic capability to fuel acetogenic metabolism with l-cysteine (independent of CdS), we dissected its metabolisation using resting cells of *M. thermoacetica* grown without cadmium. This time, glucose-pregrown cultures were harvested in the exponential growth phase, as these cells are most metabolically active and usually used to assess metabolic processes. The resting cells were incubated with d- or l-cysteine or without an energy source. As apparent from Fig. 3A, acetate was produced from l- but not d-cysteine, but cells also produced acetate from internal storage compounds (236 ± 28, 106 ± 11, 165 ± 38 µM/h and 8.9 ± 1.4, 4.1 ± 0.4, 6.9 ± 2.4 mM). CO_2_/CO_3_^2−^ was not required for acetate formation from l-cysteine (269 ± 41 µM/h; 9.2 ± 1.6 mM) (Fig. 3A). To assess metabolic activity as a result of l-cysteine metabolisation alone, the acetate concentration values from control assays that did not receive cysteine were subtracted at each time point in the next assay (Fig. 3B). The rate and final acetate produced increased with increasing l-cysteine concentrations (233 ± 17 and 553 ± 66 µM/h and 7.7 ± 0.1 and 17.6 ± 0.1 mM acetate for 6 and 12 mM l-cysteine), as well as the NH_4_^+^ production (105 ± 32 and 163 ± 19 µM/h and 3.3 ± 1.0 and 5.6 ± 0.0 mM acetate) (Fig. 3B). This assay showed that l-cysteine led to acetate formation and ammonium was a by-product of this metabolism. Lower NH_4_^+^ than acetate levels could be a consequence of a renewed incorporation of the former. The oxidized dimeric form of l-cysteine, cystine, was also readily metabolised to acetate in the presence of H_2_ as an electron donor (535 ± 84 µM/h, 27.9 ± 6.3 mM acetate). This matches the observed cystine reductase activity measured in all crude extracts. Without H_2_ as reductant, cystine alone only led to low metabolic activity (102 ± 11 µM/h, 7.2 ± 2.3 mM acetate), which suggests that there is only a low pool of internal reducing equivalents stored to drive reduction of cystine to cysteine, which can then be further utilized for acetogenesis. In another control, H_2_ + CO_2_ alone only led to acetate formation after a 24-h-lag phase (530 ± 80 µM/h, 24.8 ± 6.4 mM acetate) (Fig. 3C). The delayed and low or missing acetate formation from H_2_ + CO_2_ in resting cells or AAP experiments can be explained by the observed inability of this strain (DSM 521) to grow well on H_2_ + CO_2_. A combination of sulfide and l-cysteine increased the initial metabolic activity compared to cysteine alone (344 ± 40 and 135 ± 13 µM/h), but the final acetate produced was only slightly increased (9.3 ± 2.5 and 7.2 ± 3.2 mM acetate) (Supplementary Fig. 7). This could be explained by (at least) one of the three sulfite reductases encoded in the genome (*MOTHE_15990-16000* [dsvB1-dsVA1], *MOTHE_16350-16360* [dsvB2-dsvA2], *MOTHE_11380-11400* [asrC1-asrB2-asrA2]) oxidizing sulfide to sulfite. This reaction liberates six electrons which could be used to drive CO_2_ fixation to yield an additional 0.75 mol acetate. However, sulfide + CO_2_ was not supportive of metabolism above background activity, indicating that sulfide alone is not sufficient to drive this reaction. Overall, the resting cell experiments clearly showed that l- but not d-cysteine was metabolised to acetate, NH_4_^+^ was a byproduct and cystine (the oxidized dimeric form of l-cysteine) can also be metabolised to acetate when enough reducing power is present (for example in the form of H_2_).

### NMR tracing experiments with ^13^C-l-cysteine

To trace the metabolisation of carbon from l-cysteine, we performed resting cell experiments with l-cysteine-1-^13^C (^13^C-l-cysteine). Subsequent ^1^H-NMR analyses only showed a minor increase from 1.08% ^13^C-acetate of the control (commercial acetate) to 1.56 or 1.63% ^13^C-acetate in the resting cells exposed to 6 or 12 mM ^13^C-l-cysteine after 24 h of incubation (Supplementary Fig. 8). This indicates that ^13^C-l-cysteine is converted to pyruvate and the subsequent decarboxylation of pyruvate to acetyl-CoA by the PFOR generates ^13^CO_2_. The small increase in ^13^C-acetate in the AAP experiments is a result of ^13^CO_2_ (produced from cysteine metabolisation) fixation via the WLP.

### Transcriptomic changes in response to cysteine, cadmium or blue light

To shed light on global metabolic processes in response to cysteine, cadmium or blue light, we performed transcriptome analyses. For the identification of differentially expressed genes (DEGs) we applied a log_2_-fold change (FC) of + 2/− 2 and a p-adjust value < 0.05 as cutoff. The analyses then focussed on (a) general features and clusters showing highest DEGs, (b) the central carbon and energy metabolism of acetogens and (c) redox and energy metabolism in response to cysteine, cadmium or blue light as well as (d) the identification of genes responsible for cysteine metabolisation and transport.

#### General features and highest DEGs

**Figure 4:**
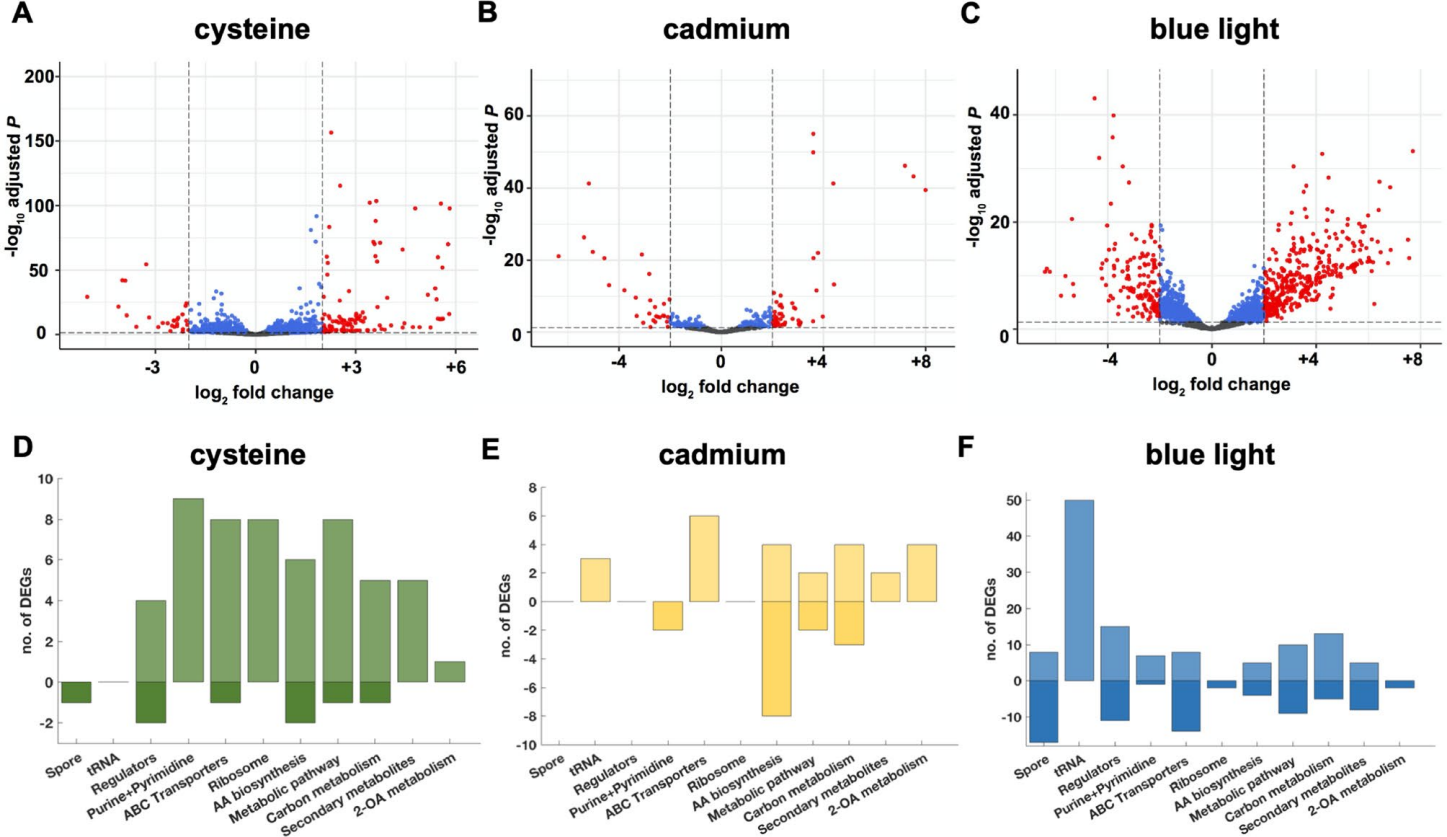
Volcano plots and KEGG categorization of DEGs in response to cysteine, cadmium or blue light. (**A**–**C**) Volcano plots depict all 2594 genes. An FC of + 2/− 2 and a p-adjust value > 0.05 was used as cutoff. Red, genes meeting these criteria (DEGs); blue, genes meeting the p-adjust value; grey, genes with no significant differential expression. (**D**–**F**) Number (no.) of DEGs annotated in selected KEGG pathways. *AA* amino acid, *2-OA* 2-oxocarboxylic acid.

Out of 2594 total genes 117, 53 or 378 genes were upregulated and 28, 28 or 151 were downregulated in response to cysteine, cadmium or blue light (Fig. 4A–C). Metabolic modules containing the highest DEGs under the three conditions are depicted in Fig. 4A–C. Almost half of the DEGs are connected to processes deposited in the KEGG database (Fig. 4D–F). The general trend was that cysteine led to an upregulation in metabolic processes, transport and ribosomal genes (Fig. 4D) whereas cadmium led to an upregulation of transporter genes, metabolic processes and tRNA and a pronounced downregulation of amino acid biosynthesis routes (Fig. 4D). Blue light triggered the largest changes in expression with 20% DEGs. The majority of DEGs encode tRNA, which were exclusively upregulated. Genes associated to spore formation, regulators, transporters and metabolic pathways, carbon metabolism and secondary metabolites also showed many DEGs in both directions (up- and downregulated) (Fig. 4F).

To shed more light onto the metabolic and cellular changes, we assessed the clusters with the highest DEGs (Supplementary Table 1). With cysteine, the most upregulated genes (DEGs +) encode a 2-hydroxy acid oxidoreductase and an aminotransferase (FC = + 5.8 and + 5.6) as well as a 10-gene cluster (FC = + 4.1 to + 5.8) comprising an l-cysteine desulfidase (*MOTHE_14040*). Both clusters are responsible for metabolization of cysteine and a detailed description is given below (“Redox and energy conservation”). Another highly DEG + 5-gene cluster comprises genes involved in quinone biosynthesis (FC = + 5.2 to + 5.6). The most downregulated genes (DEGs−) encode a membrane-bound acyltransferase and a membrane integral protein with a signal peptide (FC = − 3.9 and − 5.1) which may be involved in lipid metabolism; and two clusters which comprise 6 and 3 genes (FC = − 1.0 to − 4.1 and FC = − 3.2 to − 3.9) which may be involved in transport and sugar metabolism. Closer examination of the latter two clusters suggests that they link ion transport to glycolysis (Supplementary Text 1). With cadmium, the highest DEG + clusters encode arginine and ornithine biosynthesis machinery (FC = + 6.9 to + 8.0 and FC = + 2.1 to + 4.3) and a monosaccharide ABC transporter (FC = + 1.7 to + 4.4). The highest DEGs− encode a Cd^2+^/Zn^2+^-exporting ATPase and a regulator (FC = − 4.4 to − 6.4), a cluster encoding a glycerol dehydrogenase and a NitT/TauT family transport system (FC = − 4.6 to − 5.4) and an 8-gene cluster encoding proteins involved in histidine metabolism (FC = − 1.4 to − 3.8). The role of the ATPase may be to import Cd^2+^, which is inhibited in the presence of cadmium. The ABC transporter and histidine may be linked to a potential transport of Cd^2+^ as well. After all, Cd^2+^ can also enter bacterial cells via energy-dependent manganese or magnesium transport systems, for example^14,15^ and histidine plays an important role in metal resistance in yeasts^16^ and metal transport in bacteria^17^. With blue light, the highest DEG+ clusters encode a glutamate 2,3-aminomutase with an adjacent motility protein B (FC = + 7.7 and + 4.6). The former can form β-glutamate as an (osmo)protectant^18^ against damage induced by blue light. At the same time, glutamine synthesis from glutamate was highly repressed (*MOTHE_12770*; FC = − 4.3), leading to higher pools of glutamate available for osmoprotective purposes. The NADP^+^-reducing hydrogenase was also highly upregulated (FC = + 3.7 to + 7.6) and its role could be to get rid of excess electron as H_2_. Furthermore, a glycine betaine/carnitine/choline ABC transporter was upregulated (FC = + 3.7 to 7.6). This indicates a potential uptake of glycine betaine (GB) under light stress and GB can fulfil a dual role of an osmoprotectant and energy and carbon source^19^. Among the highest DEG− was the gene product catalysing the last step of menaquinone biosynthesis (demethylmenaquinone methyltransferase) and an adjacent cation transporter (FC = − 2.9 to − 6.4), as well as an Fe^3+^-complex transport system and a dehydrogenase resembling a molybdenum-formylmethanofuran dehydrogenase (Fmd) subunit, which catalyses the reduction of CO_2_ and conversion of formate into formyl-methanofuran in methanogenic archaea^20^ (FC = − 5.3 to − 5.8). Moreover, genes encoding sporulation proteins were downregulated (FC = − 2.4 to − 5.4). Since many spore-associated genes were also upregulated (Fig. 4F) it can be deduced that sporulation of *M. thermoacetica*^21^ and possibly other Clostridia is majorly affected by blue light.

#### Central carbon and energy metabolism

**Figure 5:**
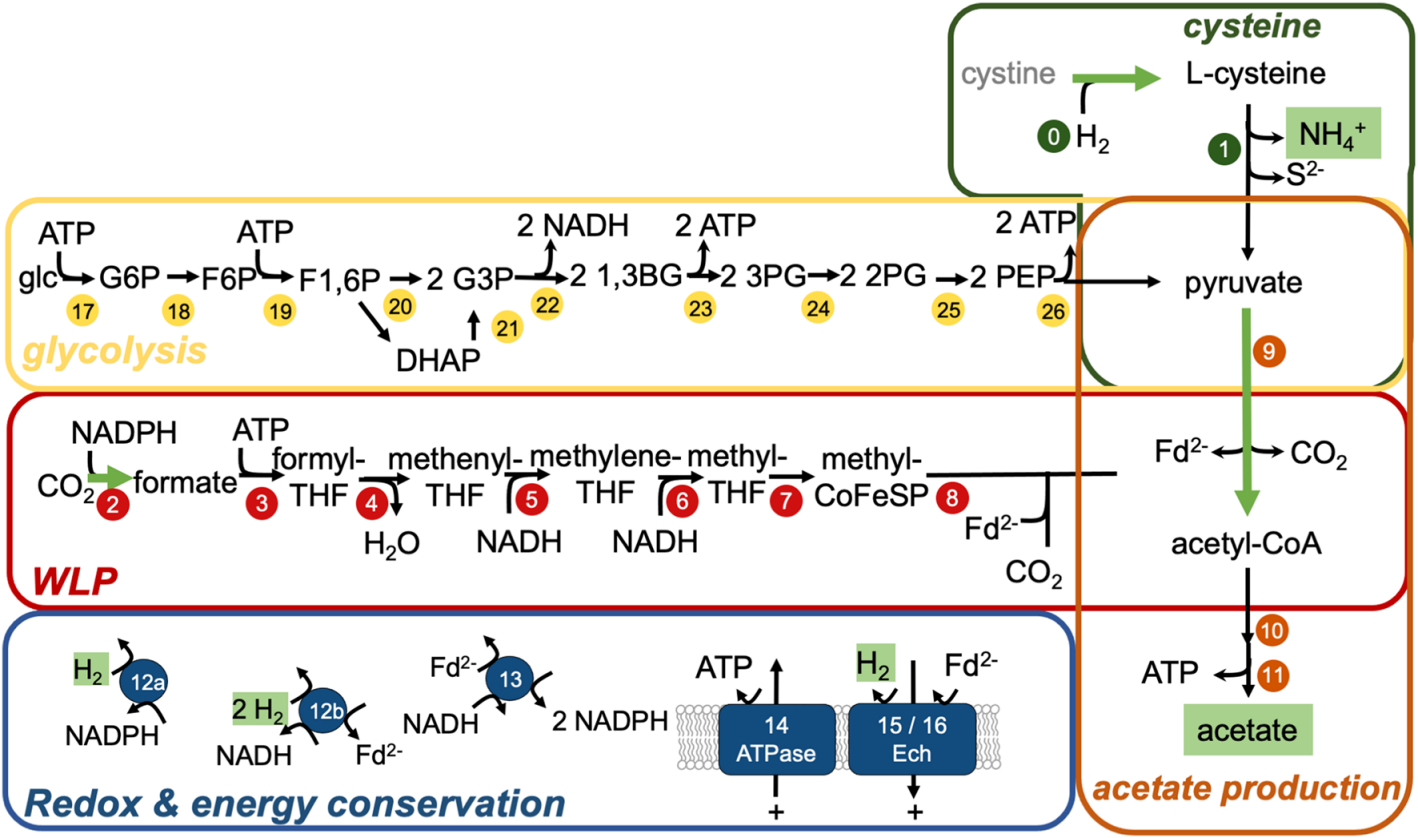
Cysteine degradation is linked to key metabolic modules in the acetogen *M. thermoacetica*. Green, cysteine module; red, WLP module; orange, acetate synthesis module; blue, redox and energy conservation module; yellow, glycolysis module. Highlighted in green are enzyme activities or metabolites that were quantified in this study. 0, cystine reductase; 1, cysteine desulfidase; for all other enzyme names see Supplementary Tables 2, 3 and 4.

The genes encoding the glycolytic pathway were unaffected by cysteine, cadmium or blue light, except for the phosphofructokinase gene which was upregulated with cysteine and blue light (both FC = + 2.6) (Fig. 5, no. 19) (Supplementary Table 2). There were also not many DEGs encoding the WLP enzymes, with a few exceptions (Supplementary Table 3). The formate-THF ligase (Fig. 5, no. 3) was upregulated with cysteine (FC = + 2.1). The formate dehydrogenase (FC = + 3.1 to + 3.6) and two PFOR gene clusters were upregulated with blue light (FC = + 2.0 to + 3.2 and + 2.1 to + 2.2), whereas the acetate kinase was downregulated (FC = − 2.6). The same expression profiles of the PFOR-encoding genes and the observation that PFOR activity was only at ~ 30–40% in CEs of CdS cells compared to normal cells suggests that cadmium inhibits the enzyme. For further analyses of the WLP see Supplementary Text 2.

#### Redox and energy conservation

The respiratory circuit of *M. thermoacetica* is made up of an F_1_F_O_ ATP synthase and two potential energy converting hydrogenases^22^. Cysteine or cadmium did not lead to DEGs within the respective *atpase*, *ech1* or *ech2* (Fhl) and associated *hyp* clusters (Supplementary Table 4). Blue light however led to a slight downregulation of a few genes within the *atpase*, *ech1* and *ech2* cluster, suggesting a decreased role of the chemiosmotic mechanism in response to blue light (FC = − 2.2 to − 2.6). This may be a protective strategy to prevent the formation of reactive oxygen species (ROS), which tend to be formed at respiratory enzymes.

Moreover, the organism possesses cytochromes and a menaquinone which make up the electron transport chain and may be involved in the chemiosmotic gradient as well^23^. As described above, genes encoding machinery for quinone biosynthesis were upregulated with cysteine but downregulated with blue light (Supplementary Table 1). Interestingly, cytochrome-associated genes (*MOTHE_21490-21500*^22^) showed no DEGs with cysteine or cadmium, but the cytochrome bd-I ubiquinol oxidase subunit 2 was significantly upregulated with blue light (FC = + 4.0). We speculate that quinones are related to electron transport whereas cytochromes are tied to oxidative stress. On top of the hydrogenases found within the *ech* clusters, *M. thermoacetica* possesses three soluble hydrogenases: the transhydrogenase (Nfn), which catalyses the simultaneous reduction of NAD^+^ and NADP^+^ from H_2_ (step 13), the NADP^+^ reducing hydrogenase (Fig. 5, no. 12a), which catalyses NADP^+^ reduction from H_2_, and the electron-bifurcating hydrogenase (HydABC), which couples the simultaneous oxidation of Fd^2−^ and NADH to H_2_ formation (Fig. 5, no. 12b). The *nfn* genes were not differentially expressed, but the NADP^+^ reducing hydrogenase was significantly upregulated with blue light (up to FC = + 7.6; one gene was also + 2.0 with cysteine) and *hydABC* was slightly downregulated with cysteine (FC = − 1.7 to − 2.2) but upregulated with cadmium and blue light (FC = + 2.2 to + 2.3/+ 2.1 to + 2.2). For more information see Supplementary Text 3.

#### Cysteine metabolisation and transport

**Figure 6:**
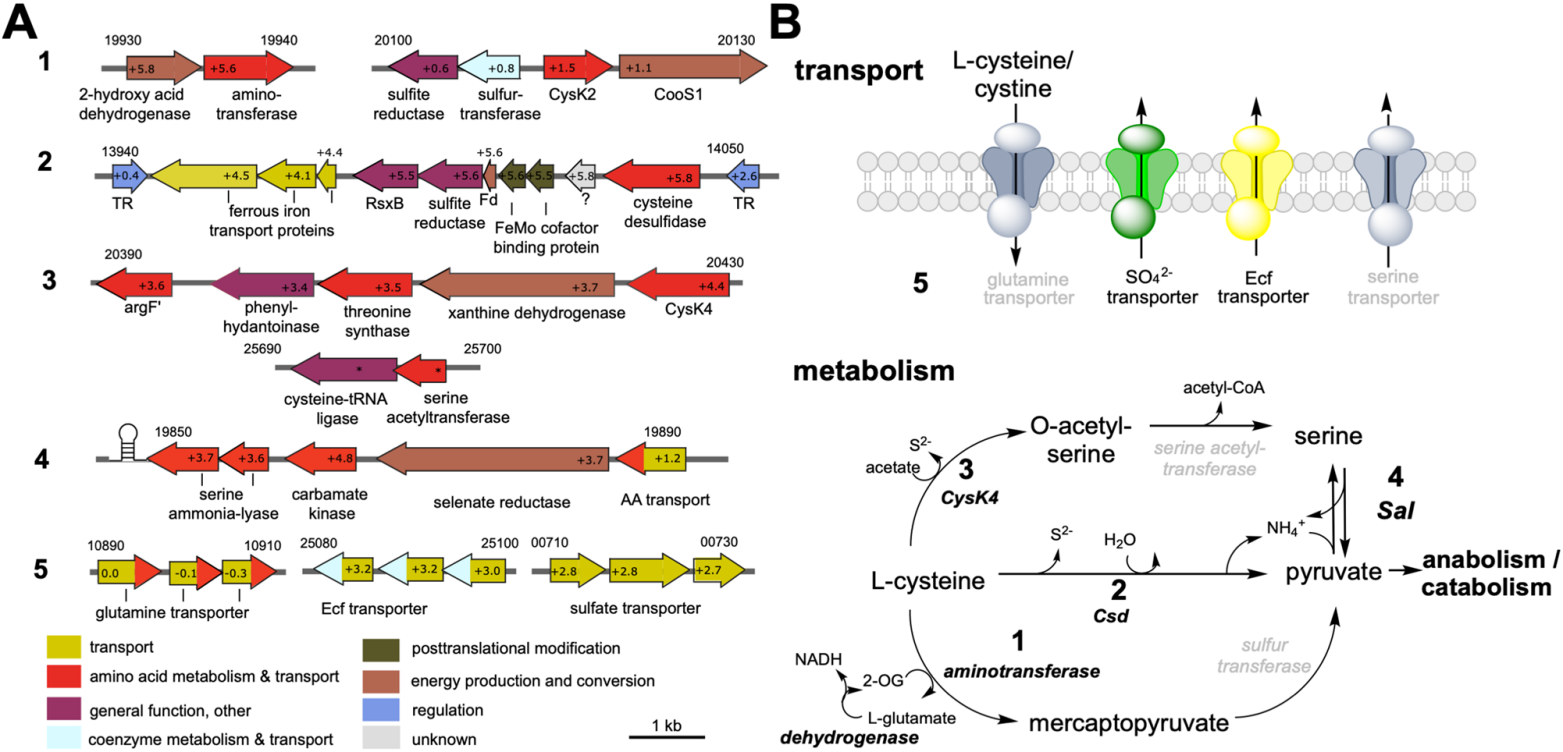
Transport and metabolisation of cysteine in *M. thermoacetica*. (**A**) Genes upregulated in cysteine cells which encode proteins that are involved in cysteine and sulfur metabolism. Colouring of genes according to PFAM classification with slight modifications. Numbers in genes indicated the log2fold change; *p-adjust value > 0.05. Numbers on top of genes indicated the locus tags (*MOTHE_*). (**B**) Proposed metabolic pathways for l-cysteine conversion. *Csd* cysteine desulfidase, *CysK4* cysteine synthase, *Sal* serine ammonia-lyase. Black or grey font indicates proteins encoded by genes that were upregulated or constitutively expressed.

Since cysteine is a central compound involved in many cellular processes such as redox homeostasis, sulfur metabolism and protein biosynthesis, it is not surprising that it can be metabolised by a range of different enzymes (Supplementary Table 5). Before the genome was screened for enzymes that can metabolize cysteine, a first route for cysteine metabolisation was already uncovered by looking at the highest DEGs in response to cysteine (“General features and highest DEGs”). They encode a hydroxy acid oxidoreductase and adjacently encoded aminotransferase, which carry out fermentation of amino acids in Clostridia via the Stickland reaction^24,25^, suggesting that *M. thermoacetica* transfers the amine group from l-cysteine onto 2-oxoglutarate (2-OG) by the aminotransferase, producing mercaptopyruvate and glutamate (Fig. 6, no. 1). The dehydrogenase then replenishes 2-OG by oxidizing glutamate and producing NADH. The mercaptopyruvate could be further converted to pyruvate by a sulfurtransferase (e.g. *MOTHE_20110*).

Subsequent analyses of the genome revealed three other types of enzymes that can metabolize cysteine: 7 carbon–sulfur lyases (Csl; EC. 4.4.1.x), which oxidize cysteine to pyruvate, liberating sulfide and ammonia (Fig. 6, no. 1); 14 sulfurtransferases which transfer the sulfur from cysteine onto an acceptor (EC. 2.8.1.x); and three PLP-dependent cysteine synthases (CS) which convert l-cysteine and acetate to *O*-acetyl-l-serine and H_2_S (belonging to EC 2.5.1.x in Supplementary Table 5). Out of the Csl encoding genes, only the l-cysteine desulfidase (Csd) gene was highly upregulated (*MOTHE_14040;* FC = + 5.8; Fig. 6 A, no. 2). Csd shares 23.6% sequence identity with the biochemically characterized homologue from the archeaon *Methanocaldococcus jannaschii*^26^, and possesses 25 of the 28 conserved amino acids from the MJ1025 encoded Csd (Supplementary Fig. 9), strongly suggesting that it depicts the second route for l-cysteine metabolisation (Fig. 6, no. 2; for further analyses see Supplementary Text 4). Furthermore, only one sulfurtransferase was upregulated with cysteine: an annotated biotin synthase (*MOTHE_17460*; FC = + 3.1), which is in fact HydE and involved in maturation of iron-only hydrogenases^27^. The three encoded CS enzymes are CysK1*,* CysK2 and CysK4. Neither *cysK1*, which is induced under oxidative stress^28^, nor *cysK2* were DEGs in cysteine cells, but *cysK4* was highly upregulated (FC = + 4.4) (Fig. 6, no. 3). Further DEGs+ encode the l-serine ammonia-lyase (Sal; FC = + 3.7 and + 3.6), which catalyses the reversible conversion of pyruvate and ammonium to serine (Fig. 6, no. 4). Interestingly, it is preceded by a glycine riboswitch, followed by two genes encoding a carbamate kinase, which makes carbamoylphosphate from CO_2_, NH_3_ and ATP, a glutamate synthase/selenate reductase and an amino acid permease, which could export excess serine (Fig. 6 no. 5). We also particularly screened for a potential uptake system for cysteine. Under aerobic conditions, cysteine autooxidizes to cystine and this dimeric form of cysteine reaches the periplasm from the extracellular space via porins. From there, many organisms rely on the ion-driven importer TcyP (YdjN) and the ATP-driven importer TcyJLN (FliY-YecSC)^29^. However, BLAST analyses (cutoff E-value 1e−5) did not reveal homologues in *M. thermoacetica* for the former and the glutamine transporter (*MOTHE_10890-10910*; 33/42/57% sequence identity) for the latter, which is also the proposed cysteine transporter in *C. difficile*^30^ (CD2177-2174 and 2172; ~ 30% sequence identity). Since it was not a DEG, it must take up l-cysteine/cystine constitutively. DEGs + encoding ABC transporters were an energy coupling factor (Ecf) transporter (*MOTHE_25080-25100*; FC = + 3.0 to + 3.2) which can transport a range of substrates including vitamins (possibly S-containing biotin)^31^, and the only sulfate transporter annotated in the genome^22^ (PBP protein/CysW/CysA; *MOTHE_00710-00730*; FC = + 2.7 to + 2.8), whereas a phosphate binding protein PtsS was downregulated (*MOTHE_04110*; FC = − 4.1). Thus, f we propose that cysteine is imported constitutively by the glutamine transporter and an upregulation of transporters for sulfur containing compounds (biotin, sulfate) then protects the cell from oxidative damage caused by excess cysteine (Fig. 6, no. 5).

## Discussion

The most appealing aspect about the CdS-*M. thermoacetica* biohybrid system which sparked us to investigate it, is its reported ability to harness light energy to drive CO_2_ fixation^3^. There are now several new publications that build on this system and have developed it further^32–34^. In our AAP experiments, we did not see a dependence of metabolism on light. However, we saw a stimulation of light on metabolic activity to some extent (up to 1.5-fold), but this was equally true for CdS and normal cells. This stimulation may be explained by light energy having an influence on enzymatic cofactors as described previously^35^. We also saw that an energization phase with H_2_ + CO_2_ resulted in significantly more acetate formation in both CdS as well as control cells. This is the unsurprising consequence of the build-up of compounds from H_2_ + CO_2_ (such as formate), stocked up pools of NADH and Fd^2−^ through the electron-bifurcating hydrogenase and because with H_2_ as reducing power, the cystine reductase, which we detected at high activities in crude extracts of CdS and control cells, can make cysteine available again from cystine. In all our AAP experiments we clearly saw a dependence of metabolism on cysteine, a common reducing agent found in all described solar-powered biohybrid systems^3,33^.

Our subsequent experiments with resting cells then revealed that l- but not d-cysteine is metabolised to acetate and increasing l-cysteine levels resulted in increased acetate and ammonium concentrations, which is sensible as biology majorly relies on the l-form of amino acids. This metabolisation was not dependent on CO_2_, demonstrating that acetate was majorly derived from a multi-carbon skeleton rather than CO_2_ fixation.

Our work demonstrates that l-cysteine is an energy and carbon source that contributes to metabolic activity of autotrophic microorganisms. Since many cultivation media for anaerobes contain l-cysteine as reducing agent in the mM range, its role as energy and carbon source must from now on be critically considered. This is particularly important when analysing autotrophic microorganisms in terms of fundamental metabolic questions, but also industrial applications. This problem may be circumvented by using Na_2_S as reducing agent and sulfur source instead of cysteine.

**Figure 7:**
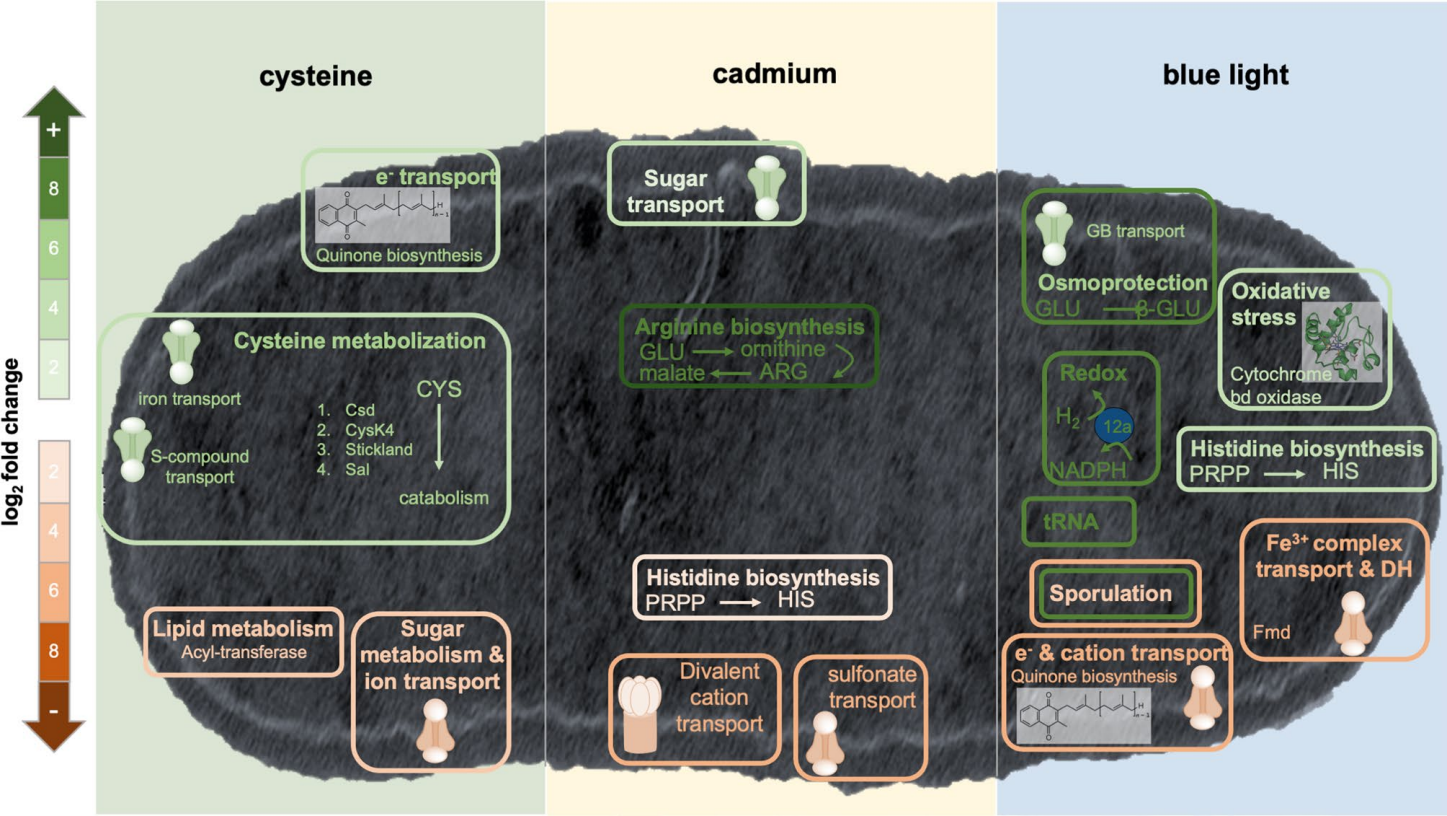
Summary of metabolic and cellular modules turned on or off by the key factors of the biohybrid systems. Depicted are cellular and metabolic modules that showed the highest differential expression patterns in response to cysteine, cadmium or blue light. For detailed description, see text.

The transcriptome analyses revealed three enzymatic paths that are employed by *M. thermoacetica* to use l-cysteine as carbon and energy source to drive metabolism and simultaneously protect the cell against its potential toxic effects (e.g. enzyme inhibition or oxidative stress^36,37^) (Figs. 6, 7). That *M. thermoacetica* does not grow on l-cysteine (this study,^11^) although it can be metabolised could be explained by an inhibition or inactivation of proteins required for growth. It is also feasible that *M. thermoacetica* can grow on l-cysteine in conjunction with a second suitable amino acid by carrying out the Stickland reaction, a trait well described in the model organism *Clostridium sticklandii* and other Clostridia^38^, but not described in acetogens so far. That quinone biosynthesis genes were also upregulated in response to cysteine abundance is another interesting aspect. Experiments with membranes of *M. thermoacetica* have indicated that menaquinone-7 acts between the two *b*-type cytochromes in an electron transport chain^23^ and the involvement of these membrane-integral electron carriers in vectorial ion transport would be possible. Thus, cysteine metabolisation may be linked to the electron transport chain and concomitantly the chemiosmotic gradient, but this remains to be investigated.

That CdS cells produced double the amount of acetate compared to normal cells was surprising and thus we examined the transcriptomic data again to seek an explanation for this. The highest DEGs in CdS cells were genes that are responsible for reversibly converting glutamate to arginine (Fig. 7). This conversion requires 4 ATP, NADPH, glutamate (two per arginine), acetyl-CoA, aspartate and bicarbonate and produces 2-OG, acetate, pyrophosphate and fumarate, which is further converted to malate (Supplementary Fig. 10). Cells may turn on this pathway because its product(s) arginine or malate (or possibly ornithine, fumarate, glutamate) chelates Cd^2+^, thus protecting enzymes from Cd^2+^ binding to sulfhydryl groups, leading to inactivation. A protective effect of arginine against cellular damage from cadmium has been described in livers of rats^39^. Moreover, PFOR, FDH and hydrogenase activities measured in crude extracts of CdS cells were decreased which may be a consequence of enzyme inhibition due to traces of Cd^2+^ entering the cell and replacing the native metal in the catalytic centre, particularly during folding of the proteins^40^. After all, the WLP relies heavily on metal chemistry and traces of Cd^2+^ could significantly interfere with almost every step of the pathway. Producing arginine requires plenty of ATP, but this is abundantly available from SLP during growth on glucose. However, when CdS cells were shifted into photosynthetic conditions, where energy and carbon sources are scarce, the intracellular pool of metabolite (arginine, malate) was converted back to channel reducing equivalents and carbon skeletons into the central WLP to make acetate. This is corroborated by the recent discovery that arginine can boost growth of the acetogen *Clostridium autoethanogenum* since it is degraded via the arginine deiminase pathway (ADI) to give rise to more acetate and ATP^41^. Therefore, the elevated metabolic activity by CdS cells results from a combination of depleting l-cysteine, the intracellular metabolite pool and autotrophic activity when H_2_ + CO_2_ is available.

Herein we also discovered that blue light, the key component of the photosynthetic biohybrid system, highly affected intracellular processes leading to a differential expression of 20% of all genes. The data uncovered protective strategies against osmo stress: a transporter of compatible solutes, production of β-glutamate; and against redox stress: a manifold increase of NADP-reducing hydrogenase and a cytochrome bd terminal oxidase. Moreover, the dramatic upregulation of tRNA indicates that tRNA abundance may be used as an additional regulatory mechanism to adjust the synthesis of specific proteins required under stress conditions, a mechanism recently discovered in yeast^42^. Furthermore, the observation that sporulation genes were majorly effected by blue light is of high interest for further exploration, since Clostridial spores are a prominent curse for industrial processes^21^ and of potential blessing for humankind’s space expansion.

Overall, this work demonstrates that the CdS-*M. thermoacetica* biohybrid system makes acetate from l-cysteine, H_2_ and intracellular storage compounds and this process was not dependent on light in our experiments. It also uncovers how l-cysteine is metabolised and reveals new intracellular processes linked to cysteine, cadmium and light. Most importantly our results demonstrate that the routine use of cysteine as a reducing agent in anaerobic cultivations and experiments must be employed with caution, as it can greatly affect and even falsify the interpretation of experiments.

## Methods

### Organisms and cultivation

*Moorella thermoacetica* (DSM 521) or *Thermoanaerobacter kivui* (DSM 2030) was cultivated at 55 or 60 °C under anaerobic conditions in complex medium that was prepared using the anaerobic techniques described previously^43,44^. The components of the complex media are listed in^3^ or^45^. Growth experiments were performed in 120-ml serum bottles (Glasgerätebau Ochs, Bovenden/Lenglern, Germany) containing 50 ml complex medium containing the normal 3.1 mM l-cysteine hydrochloride monohydrate (l-cysteine). d-glucose or additional l-cysteine were supplemented from 2.0 or 1.2 M freshly-prepared (l-cysteine) anaerobic and autoclaved stock solutions to a final concentration of 10, 25 or 50 mM d-glucose and 20 mM l-cysteine. To induce CdS nanoparticle formation, cultures were supplemented from a sterile filtered, anaerobic 100 mM stock solution of CdCl_2_ to a final concentration of 1 mM after 24 h of growth and further incubated under constant shaking at 100 rpm. Growth was routinely monitored by measuring the optical density at 600 nm (OD_600_). For experiments involving cadmium, cells were also counted using a Thoma counting chamber (0.01 mm × 0.0025 mm^2^, Hecht Assistent, Germany).

### Preparation of AAP and resting cell experiments

*M. thermoacetica* or *T. kivui* was cultivated in 1-l flasks (Schott AG, Mainz, Germany) in the above-mentioned growth media. Cells were cultivated with 50 or 25 mM d-glucose for *M. thermoacetica* or *T. kivui* and harvested in the early stationary or exponential phase for AAP or resting cell experiments, respectively. This corresponded to 72 or 48 h of growth with OD_600_s of ~ 1.0 or ~ 0.6. Cells were centrifuged (11,500×*g*, 10 min, 4 °C), and washed twice with AAP medium (0.40 g/l NaCl, 0.40 g/l NH_4_Cl, 0.33 g/l MgSO_4_ × 7 H_2_O, 0.05 g/l CaCl_2_, 0.25 g/l KCl, 0.64 g/l K_2_HPO_4_, 2.50 NaHCO_3_, 10 ml/l trace element solution DSM 141, 10 ml/l vitamin solution DSM 141) or cell suspension buffer (50 mM imidazole [pH 7.0], 50 mM KHCO_3_, 20 mM MgSO_4_, 20 mM KCl, 20 mM NaCl, 4 mM dithioerythritol (DTE), 4 µM resazurin). After the last centrifugation step, cells were resuspended in 2–3 ml of the same buffer and kept in a gas-tight Hungate tube. The protein concentration was determined according to^46^.

For AAP experiments, cells were diluted to a final concentration of 1 mg/ml in AAP medium and incubated in 120-ml serum bottles in a N_2_ + CO_2_ (80:20 [v/v]) or H_2_ + CO_2_ (80:20 [v/v]) gas atmosphere. Assays were supplemented with 6 mM l-cysteine and one set of biological triplicates was wrapped in aluminium foil and parafilm, the other reference set was not. Both triplicates were incubated in a shaking water bath at 55 °C (or 60 °C for *T. kivui*) at level 4 (G-76 Gyrotory, New Brunswick Scientific, New Jersey, USA) and exposed to royal blue light (LED Light Source SL 3500; Photon Systems Instruments, Brno, Czech Republic) with a blue light peak at 440 nm, a spectrum from 410–480 nm and a measured photon flux of 78 × 10^17^ photons m^−2^ s^−1^ [= 13 μEinstein], whereas the other triplicate was protected from light by the aluminium foil. Assays received 500 (AAP1) or 250 µl (AAP2) chemically synthesized CdS nanoparticles at the beginning of the AAP experiment (equating to a calculated final concentration of 1 or 0.5 mM CdS). 400 µl samples were withdrawn at the time points indicated, centrifuged (12,000×*g*, 2 min, 4 °C) and the supernatant was stored at − 20 °C.

For resting cell experiments, cells were diluted to a final concentration of 0.5 or 1.0 mg/ml in cell suspension buffer (cell suspension buffer for NMR contained 10 mM potassium phosphate instead of 50 mM imidazole) and incubated in 120-ml serum bottles in a N_2_ + CO_2_ (80:20 [v/v]) or H_2_ + CO_2_ (80:20 [v/v]) gas atmosphere. After a pre-incubation at 55 °C in a water bath for 15 min, assays were supplemented with 3, 6 or 12 mM l-cysteine from a freshly-prepared anoxic 300 mM stock solution, 6 mM cystine from a 300 mM stock solution (1 M solved in 1 M HCl and diluted with deionized water) or 6 mM Na_2_S from a 300 mM stock solution. ^13^C-l-cysteine (l-cysteine-1-^13^C 99 atom % ^13^C, 98% (CP); MW: 122.2 g/mol), l-cysteine (l-cysteine hydrochloride monohydrate; reagent grade, ≥ 98%; MW: 175.6 g/mol) and d-cysteine (d-cysteine hydrochloride monohydrate; reagent grade, ≥ 98%; MW: 175.6 g/mol) was purchased from Merck (Darmstadt, Germany). 400 µl samples were withdrawn at the time points indicated, centrifuged (12,000×*g*, 2 min, 4 °C) and the supernatant was stored at − 20 °C.

### Metabolite analyses via high-pressure liquid chromatography

Metabolite analyses were performed with an HPLC system (Elite LaChrom, VWR, Pennsylvania, USA) equipped with an organiser, a diode array detector (L-2455), a thermostatted column oven (L-2300), an autosampler (L-2200) and a pump (L-2130). An Acclaim organic acid column (5 μm, 4.0 × 150 mm, Thermo Scientific, USA) was used to separate acetate from other analytes. HPLC carrier solvent (100 mM Na_2_SO_4_, pH 2.65/H_2_SO_4_) and MQ-water was prepared just prior the analyses and filtered through a membrane filter (NC45-0.45 μm, Whatman, UK). Samples were diluted 1:10 with carrier solvent at a final volume of 600 μl and adjusted to a pH 2 by adding 6 μl 20% H_2_SO_4_.

To remove particles, the samples were centrifuged (16,000×*g*, 5 min, 4 °C) and the supernatant was transferred into HPLC-vials. 10 mM solutions of lactate, acetate, pyruvate, l-cysteine, cystine and formate served as standards and a sample containing only medium served as a control. 10 μl samples were applied onto the 30 °C-thermostatted column at a flowrate of 0.6 ml × min^−1^ and a run time of 15 min. The detection of the organic acids occurred at 210 nm.

### Preparation of crude extracts

*M. thermoacetica* was grown in 500 ml complex medium on d-glucose with or without 1 mM CdCl_2_ (supplemented after 24 h of growth) until the exponential or stationary growth phase and harvested at and OD_600_ of 0.6–0.7 or 1.1–1.2. All steps were carried out in an anaerobic chamber (Coy Laboratories, Grass Lake, USA) containing an N_2_+H_2_ (95:5 [v/v]) atmosphere. Cultures were harvested anaerobically by centrifugation (11,480×*g*, 10 min, 4 °C) and washed twice in harvest buffer (50 mM Tris–HCl [pH 7.5], 20 mM MgSO_4_, 20% glycerol, 4 mM DTE, 4 µM resazurin). Cells were then resuspended in 1/10 of the initial volume of the harvested culture and supplemented with 0.1 mg lysozyme/ml cell suspension and incubated at 37 °C for 1 h under constant shaking at 100 rpm. The suspension was centrifuged as above and resuspended in 20 ml harvest buffer, supplemented with 200 µM phenylmethylsulfonylfluoride and a spatula tip of *DNase*I and cells were lysed by two consecutive passages though a French pressure cell (SLM Aminco, Co. G. Heinemann, Schwäbisch Gmünd, Germany), equipped with an anaerobic outlet cannula under “high” conditions at 1000 *psig* (120 MPa). Undisrupted cells and cell debris were separated by a final centrifugation step (11,480×*g*, 20 min, 4 °C) and the protein concentration of the supernatant containing the crude extract was measured as described previously^47^.

### Enzyme assays

All enzyme activities were measured at 55 °C in 1.8-ml anaerobic cuvettes (Glasgeräutebau Ochs, Bovenden/Lenglern, Germany) filled with enzyme buffer at a final liquid volume of 1 ml under a 100% N_2_ gas atmosphere, unless indicated otherwise. PFOR activity was measured at 670 nm in enzyme buffer 1 (50 mM Tris–HCl [pH 7.5], 10 mM NaCl) containing 10 µM coenzyme A (CoA), 10 µM thiamine pyrophosphate (TPP), 50 mM sodium pyruvate and 5 µM methylene blue (MB). Cystine reductase activity was measured at 604 nm in enzyme buffer 2 (500 mM KP_i_ [pH 8.0], 10 mM NaCl, 4 mM DTE, 4 µM resazurin) containing 0.5 mM methyl viologen (MV) and 6 mM cystine (30 µl of a 200 mM stock solved in 1 M HCl) in a H_2_ + CO_2_ (80:20 [v/v]) atmosphere. Hydrogenase activity was measured at 604 nm in enzyme buffer 2 containing 5 mM MV in a H_2_ + CO_2_ (80:20 [v/v]) atmosphere. Formate dehydrogenase activity was measured in enzyme buffer 2 at 340 nm containing 10 mM NADP^+^ and 50 mM formic acid. The extinction coefficient used to calculate specific activities were 28.5, 6.2 and 13.8 mM^−1^ cm^−1^ for MB, NADP and MV, respectively. Measurements were performed in a thermostatted spectral photometer (V-530, Jasco, Pfungstadt, Germany).

### Chemical synthesis of CdS nanoparticles

CdS nanocrystals were synthesized as described previously^48^ and the reaction was performed in a 1-l three-neck flask fitted with a septum. Specifically, 500 mL of an argon-saturated aqueous solution of 0.2 mM Cd(ClO_4_)_2_ and 0.2 mM sodium hexametaphosphate was filled into the flask under argon atmosphere and subsequently, a stoichiometric amount of H_2_S was injected into the gas phase. The solution was then stirred for 10 min, flushed with argon and the pH was adjusted to the required value of 11 by adding NaOH. Finally, 3 ml of a 0.1 M Cd(ClO_4_)_2_ solution was added dropwise while stirring and the reaction solution was aged for 24 h before use.

The CdS solution was concentrated with a 10 kDa-cutoff centricon (Amicon Ultra-15, Merck, Germany) to half the volume and readjusted to the initial volume with distilled water 3 times. Photometric analyses of the chemically synthesized nanoparticles were performed using a spectral photometer (V-530, Jasco, Pfungstadt, Germany) and a spectrofluorophotometer (RF-6000, Shimadzu, Kyoto, Japan) (Supplementary Fig. 4).

### TEM analyses

Transmission electron microscopy (TEM) was performed according to^49^. Briefly, the bacteria were fixed with 2% glutaraldehyde in cacodylate buffer (75 mM, pH 7.0) for 2 h, postfixed with 1% osmium tetroxide at 4 °C overnight. The samples were dehydrated through a series of graded acetone concentrations (30–100%) and finally embedded in plastic according to^50^. Ultrathin sections were obtained with a ultramicrotome (Ultracut E, Leica-Reichert-Jung, Nußloch, Germany) and stained with uranyl acetate followed by lead citrate^51^. Sections were viewed with a LEO 906 E TEM (LEO, Oberkochen, Germany) operated at 100 kV and equipped with the MultiScan CCD Camera (Model 794) of Gatan (Munich, Germany) using the Digital Micrograph software version 2.0.2 from Gatan to acquire, visualize, analyze, and process image data.

### NMR data acquisition and sample treatment

The samples were acquired on an AVANCEIII HD 600 MHz spectrometer (AV3600, Bruker) with the TopSpin software (version 3.2, Bruker BioSpin GmbH). The spectrometer frequency was operating at 600.13 MHz. TMSP (*δ* = 0 ppm) was used as internal standard for all samples. The chemical shifts *δ* were given in parts per million (ppm). All spectra were recorded at 300 K. The ^1^H-NMR spectra were measured with water suppression (excitation sculpting) applying 128 scans (2 dummy scans), 32,768 complex data points, a relaxation delay of 5 s and a spectral width of 9615.4 Hz. The receiver gain was set to 2050. For data processing, the FIDs were Fourier-transformed with a line broadening factor of 0.3 Hz, an automatic baseline correction and a phase correction with TopSpin (Version 3.5 pl 7, Bruker BioSpin GmbH).

The samples were measured in cell suspension buffer for NMR/deuterated buffer 90:10%. The buffer was a 400 mM water-*d*_6_ phosphate buffer. The buffer was made of 1.36 g (10 mM) potassium dihydrogen phosphate and 1.74 g (10 mM) dipotassium hydrogen phosphate in 100 mL deuterated water. 8.89 mg sodium trimethylsilylpropionate (TMSP) were added to the solution as an internal standard.

### Sampling, sequencing and analyses of transcriptomic data

The transcriptome of biohybrid cells (*M. thermoacetica*-CdS) grown on 50 mM d-glucose, supplemented with 1 mM CdCl_2_ after 1 day and harvested after 3 days of growth (early stationary phase) was compared to control cells  (without CdCl_2_ supplementation) from the same growth phase. To investigate the effect of cysteine (complex medium + 20 mM l-cysteine), the transcriptome was analysed from glucose-grown cells harvested in the mid-exponential growth phase and compared to glucose-grown cells under normal conditions (complex medium) and harvested in the same growth phase.

Harvested cells were resuspended in 800 µl RLT buffer (RNeasy Mini Kit, Qiagen) with β-mercaptoethanol (10 µl/ml) and cell lysis was performed using a laboratory ball mill. Subsequently 400 µl RLT buffer (RNeasy Mini Kit Qiagen) with β-mercaptoethanol (10 µl/ml) and 1200 µl 96% [v/v] ethanol were added. For RNA isolation, the RNeasy Mini Kit (Qiagen) was used as recommended by the manufacturer, but instead of RW1 buffer RWT buffer (Qiagen) was used in order to isolate RNAs smaller than 200 nt also. To determine the RNA integrity number (RIN) the isolated RNA was run on an Agilent Bioanalyzer 2100 using an Agilent RNA 6000 Nano Kit as recommended by the manufacturer (Agilent Technologies, Waldbronn, Germany). Remaining genomic DNA was removed by digesting with TURBO DNase (Invitrogen, ThermoFischer Scientific, Paisley, United Kingdom). The Pan-Prokaryozes riboPOOL kit v1 (siTOOLS BIOTECH, Planegg/Martinsried, Germany) was used to reduce the amount of rRNA-derived sequences. For sequencing, the strand-specific cDNA libraries were constructed with a NEBNext Ultra II directional RNA library preparation kit for Illumina (New England BioLabs, Frankfurt am Main, Germany). To assess quality and size of the libraries samples were run on an Agilent Bioanalyzer 2100 using an Agilent High Sensitivity DNA Kit as recommended by the manufacturer (Agilent Technologies, Waldbronn, Germany). The concentration of the libraries was determined using the Qubit dsDNA HS Assay Kit as recommended by the manufacturer (Life Technologies GmbH, Darmstadt, Germany). Sequencing was performed by using the HiSeq4000 instrument (Illumina Inc., San Diego, CA, USA) using the HiSeq 3000/4000 SR Cluster Kit for cluster generation and the HiSeq 3000/4000 SBS Kit (50 cycles) for sequencing in the single-end mode and running 1 × 50 cycles. Between 16.351.837 and 114.489.763 raw reads were generated for the samples (for details see Supplementary Table 6). After processing of the 50 bp single-end raw reads with trimmomatic (version 0.39)^52^, Salmon (v 1.2.1)^53^ was used for mapping of the trimmed single-end reads (Supplementary Table 6) against the genome of *M. thermoacetica* DSM 521^54^. A file containing all annotated transcripts (without rRNA genes) and the whole genome as decoy was prepared with a k-mer size of 11 as mapping backbone. Decoy-aware mapping was done in selective-alignment mode with “–mimicBT2”, “–disableChainingHeuristic”, and “–recoverOrphans” flags as well as sequence and position bias correction. For –fldMean and –fldSD, a value of 325 and 25 was used, respectively. Salmon’s quant.sf files were subsequently loaded into R (v 4.0.0)^55^ using the tximport package (v 1.16.1)^56^. Normalization of the reads was done with DeSeq2 (v 1.28.1)^57^ and foldchange-shrinkages were calculated with DeSeq2 and the apeglm package (v 1.10.0)^58^. For figures like heatmaps and PCAs, the rlog function of DeSeq2 with “blind” parameter set to FALSE was used. Most figures were created with ggplot2 (v 3.3.2)^59^, whereas volcano plots were created with the EnhancedVolcano package (v 1.6.0)^60^. For analysis based on KEGG annotation, the clusterProfiler (v 3.16.0)^61^ and pathview (v 1.28.0)^62^ packages were used. Genes with a log_2_-fold change of + 2/− 2 and a p-adjust value < 0.05 were considered differentially expressed. Genes were also manually analysed and curated using the KEGG, KO Database, the Integrated Microbial Genomes–Expert Review (IMG-ER) database, InterPro, TMHMM and BLAST. For genes related to several KEGG pathways only one category was used with the following preference: 2-OA > AA biosynthesis > Purine + Pyrimidine > Carbon metabolism > Secondary metabolites > Metabolic pathway. Genes in the categories tRNA, spore and transporters were categorized manually by annotation (‘tRNA’, ‘spo(re)’, ‘regulator/activator/repressor’).

## Supplementary Information

Supplementary Information.

## Data Availability

Transcriptomic data that support the findings of this study have been deposited in the National Center for Biotechnology Information's (NCBI) Sequence Read Archive (SRA) under accession no. SRR12440685–SRR12440713. All other data of this study are available from the corresponding author upon reasonable request.
