# Supplementary material for: Cysteine: an overlooked energy and carbon source
Source: Sci Rep. 2021 Jan 25;11:2139. doi: 10.1038/s41598-021-81103-z (PMC7835215; doi:10.1038/s41598-021-81103-z)
Supplement: Supplementary file 1 — Supplementary Information. [file 41598_2021_81103_MOESM1_ESM.docx]

**Supplementary Information for**

**Cysteine: an overlooked energy and carbon source**

Luise Göbbels^1^, Anja Poehlein^2^, Albert Dumnitch^1^, Richard Egelkamp^2^, Cathrin Kröger^1^, Johanna Haerdter^3^, Thomas Hackl^3^ , Artur Feld^4^, Horst Weller^4^, Rolf Daniel^2^, Wolfgang R. Streit^1^, Marie Charlotte Schoelmerich^1,*^

**The PDF file includes**

Supplementary Figures (Supplementary Fig. 1 – 9)

Supplementary Tables (Supplementary Table 1 – 6)

Supplementary Text (Supplementary Text 1 – 4)

Supplementary References

**Supplementary Fig. 1. Schematic representation of the proposed biohybrid system.** Cys, cysteine; CySS, dimeric oxidized form cystine. Depicted according to ^1^ and modified version of ^2^.

**
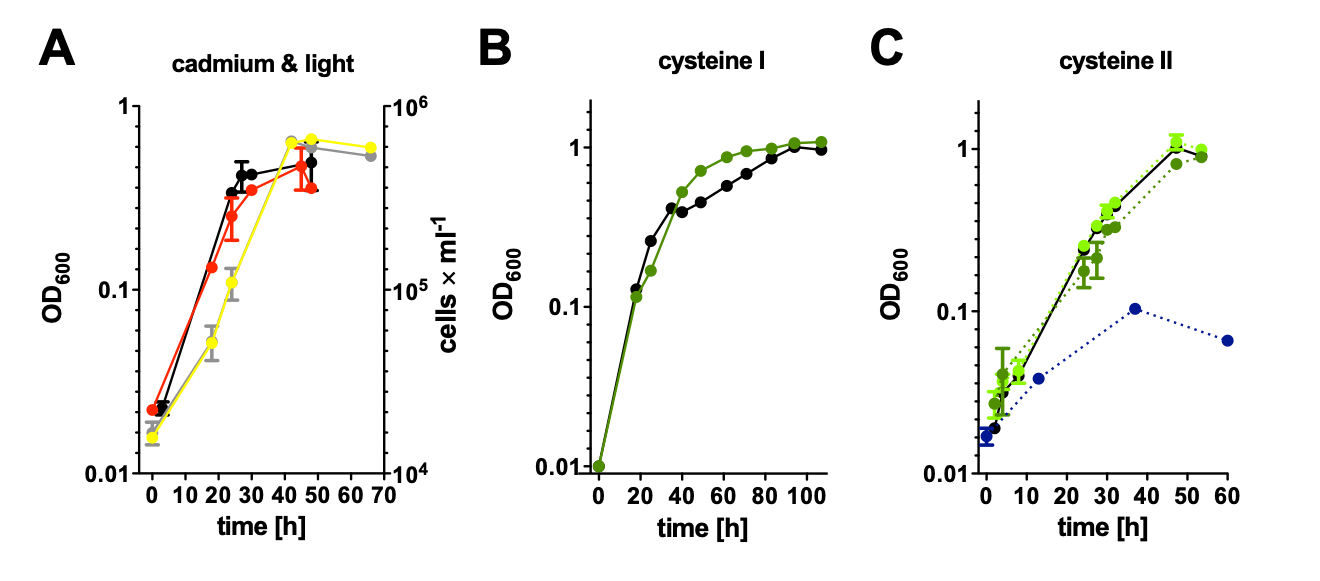
**

**Supplementary Fig. 2. Growth of *M. thermoacetica* in dependence of cadmium, light, and cysteine.** *M. thermoacetica* DSM 521 was grown in 50 ml complex medium with 50 mM D-glucose at 55°C. (A) Cells were grown under constant shaking at 100 rpm and supplemented with 6 mM L-cysteine (red, black) with (red) or without (black) 1 mM CdCl_2_ at 24 h. The other cultivations were performed under constant shaking in a water bath under blue light exposure (yellow) or in the dark (grey). (B) Cells were grown on 50 mM D-glucose with (green) or without (black) 20 mM additional L-cysteine. (C) Cells were inoculated into complex medium containing 10 mM D-glucose (black), 10 mM L-cysteine (blue) or 10 mM D-glucose + 10 mM L-cysteine (light green) or 10 mM D-glucose + 20 mM L-cysteine (dark green). N = 2, SD.


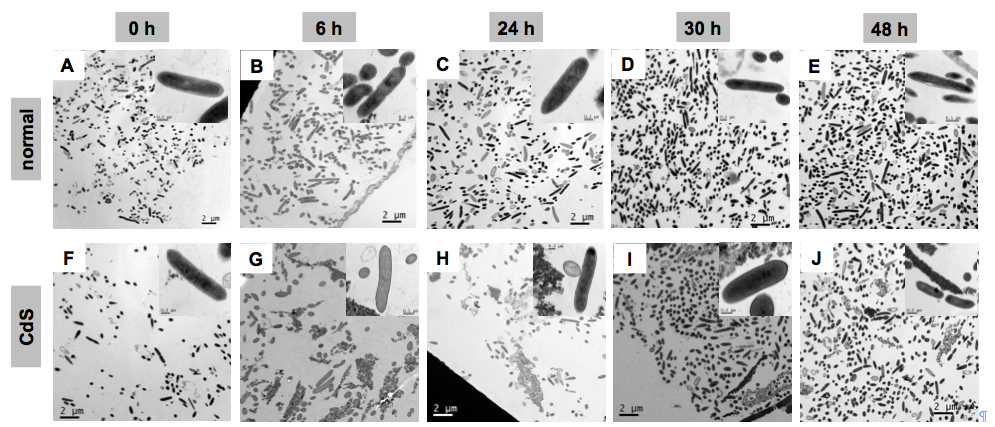


**Supplementary Fig. 3. Growing cells of *M. thermoacetica* with or without cadmium supplementation.** Cells were grown in 50 ml complex medium containing 50 mM D-glucose as carbon source. All cultures were supplemented with 6 mM L-cysteine after 24 h of growth (indicated as time point 0 h) and some cultures (F-J) also received 1 mM CdCl_2_. 800 µl cultures were centrifuged at the time points indicated and cell pellets were prepared for TEM analyses.

**
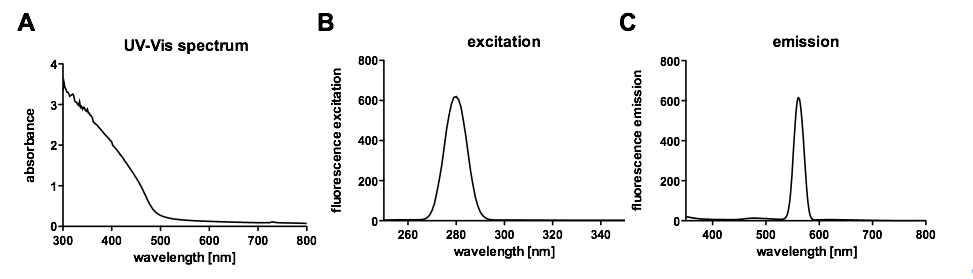
**

**Supplementary Fig. 4. Spectral properties of the chemically synthesized CdS nanoparticles.** Nanoparticles were diluted 1:10 in water. The UV-Vis spectrum was determined (A) and the excitation maximum is at 280 nm (B) and the emission maximum is at 561 nm (C).


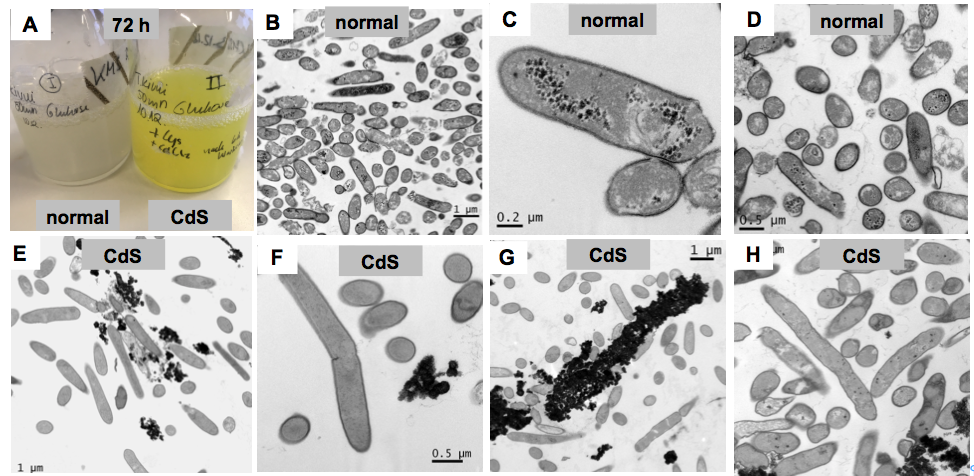


**Supplementary Fig. 5. Normal and CdS cells of *T. kivui*.** A.*T. kivui* after 72 h of growth in complex medium on 25 mM D-glucose with or without CdCl_2_ supplementation after 6 h of growth. B-D. Normal cells of *T. kivui* in photosynthetic medium after 0 h (B,C) or 58 h (D) in blue light. E-H. CdS-*T. kivui* cells in photosynthetic medium after 0 h (E, F) or 58 h (G, H) in blue light.

**
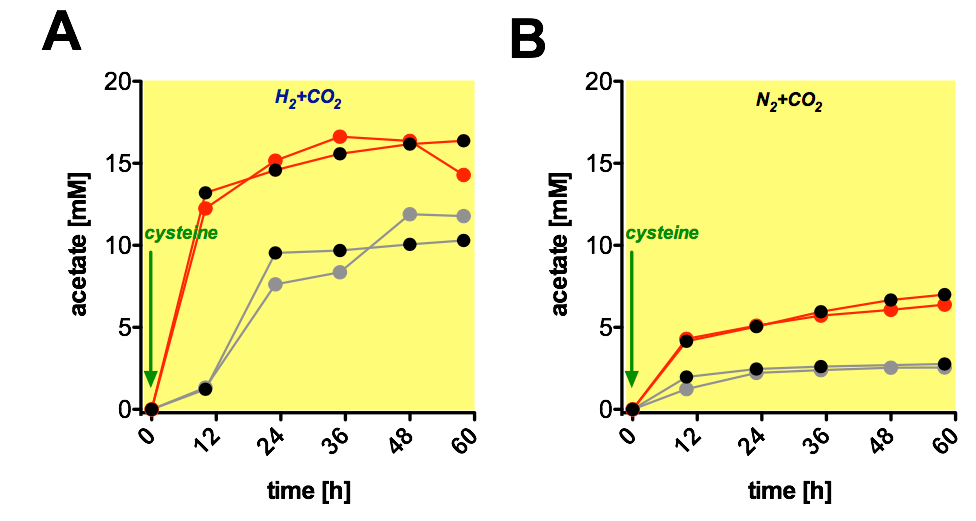
**

**Supplementary Fig. 6. Metabolic activity of *T. kivui* CdS cells or normal cells under photosynthetic conditions.** *T. kivui* cells grown on 25 mM D-glucose with (red lines) or without 1 mM CdCl_2_ (supplementation after 24 h) were harvested after 72 h and transferred into photosynthetic medium at 1 mg/ml. The assays were incubated at 60°C in a shaking water bath in blue light (coloured circles) or in the dark (black circles) in a 1.3 × 10^5^ Pa H_2_+CO_2_ (A) or N_2_+CO_2_ (80:20 [v/v]) (B) atmosphere and supplemented with 6 mM L-cysteine.

**
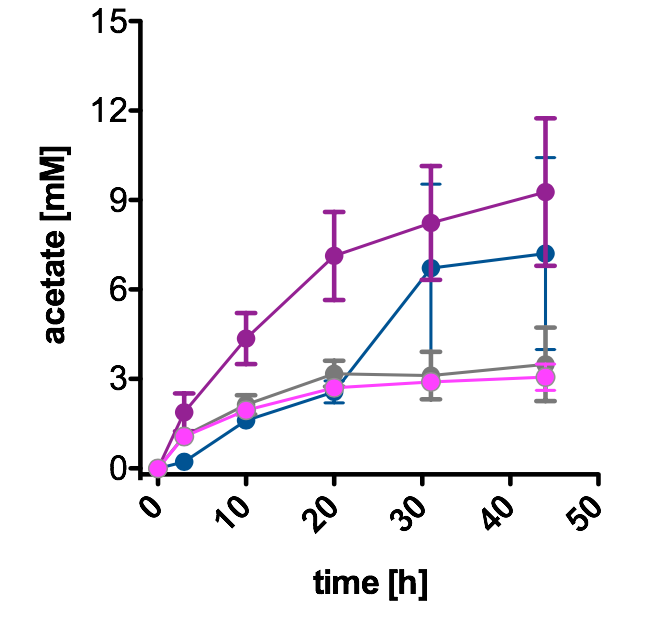
**

**Supplementary Fig. 7. Metabolic activity by resting cells of *M. thermoacetica* in dependence of sulphide.** *M. thermoacetica* was grown on 50 mM D-glucose until the exponential growth phase, cells were harvested and transferred into cell suspension buffer at a final concentration of 1 mg/ml under a 1.3 x 10^5^ Pa N_2_+CO_2_ (80:20 [v/v]) atmosphere and incubated at 55°C under constant shaking (100 rpm) in the dark. Assays were supplemented with 6 mM L-cysteine (blue), 6 mM L-cysteine + 6 mM Na_2_S (purple), 6 mM Na_2_S (pink) or none (grey).

**
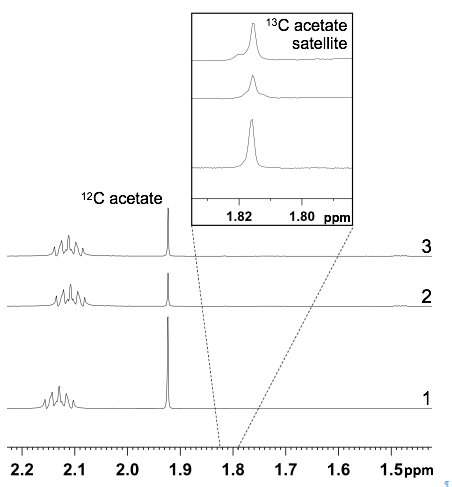
**

**Supplementary Fig. 8. ^1^H-NMR spectra (600 MHz) of resting cells exposed to 13C-L-cysteine.** The samples were measured in a mixture of 90%/10% resting cell and deuterated buffer. The spectra were recorded at 300 K and measured with water suppression (excitation sculpting). The zoom factor of the cutout was set to fourteen times higher than the initial spectra. The metabolization of ^13^C-labelled cysteine to acetate should be checked for spectra 2 and 3. For this purpose, the intensity of the ^12^C singlet of acetate (1.92 ppm) was compared to twice the intensity of the ^13^C-satellite (1.82 ppm). In comparison, the first spectrum shows an acetate sample with a natural ratio of ^12^C to ^13^C acetate (^12^C/^13^C 98.92%:1.08%). For spectrum 2, 6 mM ^13^C-cysteine and for spectrum 3, 12 mM ^13^C-cysteine was used. Spectrum 2 shows an increase in the ^13^C content of acetate (^12^C/^13^C 98.44%:1.56%). Spectrum 3 also shows an increase in the ^13^C signal of acetate (^12^C/^13^C 98.37%:1.63%).

MJ1025 MVSIMNKNELITEILKNEVVKALG**C**TEVGLIGYTVAKAKPEDLYSIKEIKLILDKGTFKN 60

MOTHE_14040 ----LLDQQTLINLLHQEADVAIGCTEPVMVALAAAKTRDMLGTLPRLVDISVSSAVWKN 56

: .:: : ::*::*. *:**** ::. :.**:: : :.: :....:**

MJ1025 AFSVGVPNTNKFGILPAVVGGLLGREENKLEVFKDIK---YDEKLEEFIENKLKIEV--I 115

MOTHE_14040 ARRVGLPGTGEKGLAMAAAMGLLAPVEAGQRLLAALTPVQVEQAKILVREGVVKVGVVAA 116

* **:*.*.: *: *.. ***. * .:: :. :: . *. :*: *

MJ1025 DSDVYCKVIIKANKVY-EAETKGSHSGK-----------SLSDDLKNAYKSLTLKDFIDY 163

MOTHE_14040 KEGLYARAVARSNQHEAIVELNGSHKNFSALWLDGRMAGGAGENLNLKLEALLAQDYQSL 176

...:*.:.: ::*: .* :***.. . .::*: ::* :*: .

MJ1025 IE----DIPEE----------VIKIIKETIETNKNLSTPEVPEDFISLDLKDEILNHML- 208

MOTHE_14040 LKQVLSLSPEELYFLYQGAEDILTFAREIHQGGRN-PLSAMASFFRRTESGGESLEVLIR 235

:: *** ::.: :* : .:* : . * : .* *: ::

MJ1025 KKTVSAVYNRMIGINKPAMAIAGSGNMGLTATLPIIAYDEIKGHDEEKLTKSITLSALTT 268

MOTHE_14040 NLTGIAVAERMAGATYPVLTCAGSGNQGILAAVSLLLAGQELRAGPESVTRALAIAHFTN 295

: * ** :** * . *.:: ***** *: *:: :: .: . *.:*:::::: :*.

MJ1025 IYSAYHSSYISAM**C**GCVNRGGIGAVSGLSYYIFG-FDRIEESIKSFTANLPGIV**C**DGGKI 327

MOTHE_14040 MYLKAYTGKLSPLCGAVTGG-AGVAAAICWLLEGSCQQIINAMQIVLGNLCCVICDGAKE 354

:* ::. :* :**.*. * *..:.:.: : * ::* :::: . .** ::***.*

MJ1025 G**C**ALKIASGVFAIYLSLFS-----KVPYTNGIVGKDFKECIENIGKIGKA--------MK 374

MOTHE_14040 SCALKISTAAVEAVRAGYMACQGINLEAGTGIVGKKLEDTMELVRKVYQGGLGEIDYYLG 414

.*****::... : : :: .*****.::: :* : *: :. :

MJ1025 PVDDEIIEILKNKK 388

MOTHE_14040 KVDYL----LSTN- 423

** *..:

**Supplementary Fig. 9. Sequence alignment of *csd* (*MOTHE_14040*) from *M. thermoacetica* against homologue from *M. jannaschii* (*MJ1025*) gene.** 25 of the 28 identical conserved amino acids (according to Tchong *et al.,* 2005) are highlighted in yellow, 3 exchanged amino acids (S264🡪A, L266🡪F and G310🡪C) are highlighted in red. The ligands for the [3Fe-4S] cluster are all present and indicated by the red font.


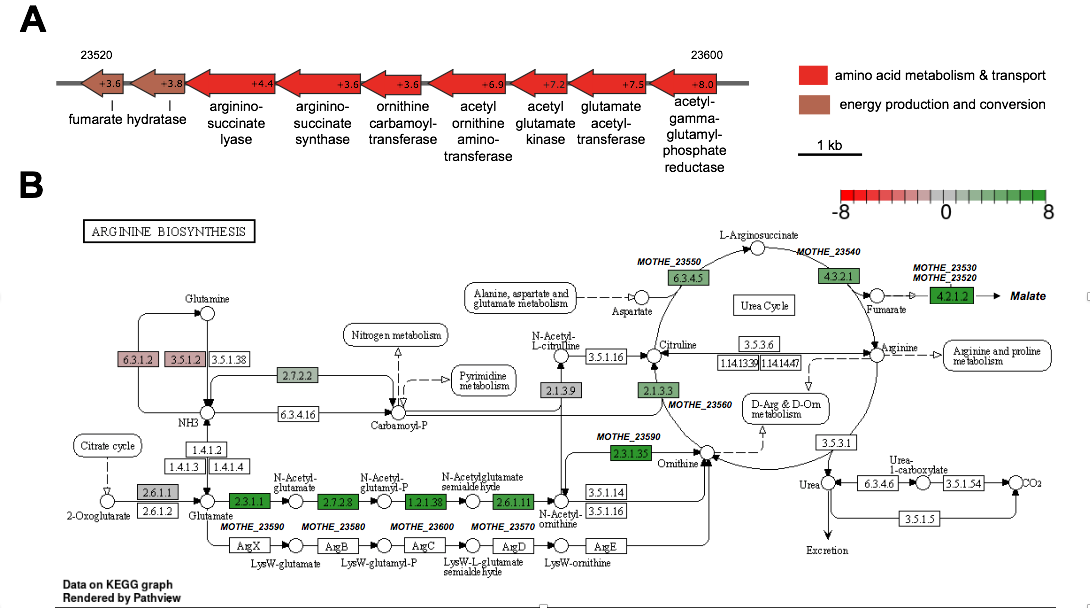


**Supplementary Fig. 10. Arginine biosynthetic cluster and metabolism route lights up in cadmium cells.** A. Highest DEGs in cadmium cells are arginine biosynthetic genes. Coloring of genes according to PFAM classification with slight modifications. Numbers in genes indicated the log2fold change. Numbers on top of genes indicated the locus tags (*MOTHE_*). B. Arginine biosynthesis pathway with log2fold change in gene expression highlighted. Panel B was constructed using pathview ^3^.

**Supplementary Table 1. Expression of gene clusters with highest DEGs in response to cysteine or cadmium.** Numbers indicate log2fold change (FC) from glucose-grown cells in the exponential growth phase with (condition 1: cysteine) or without (control 1) 20 mM L-cysteine or in the early stationary growth phase with (condition 2: cadmium) or without (control 2) 1 mM Cd^2+^ and harvested in the exponential growth phase. *, no significant differential gene. Shown are locus tags of *M. thermoacetica* DSM 521 (MOTHE) and the ATCC39073 (Moth) strain as reference.

| **locus tag** | **predicted function** | **cysteine** | **cadmium** | **blue light** |
| --- | --- | --- | --- | --- |
| MOTHE_19930/Moth_1954  MOTHE_19940/Moth_1955 | **REDOX**  putative 2-hydroxyacid dehydrogenase  aspartate aminotransferase | **+5.8**  **+5.6** | *  * | *  * |
| MOTHE_13950/Moth_1405  MOTHE_13960/Moth_1406  MOTHE_13970/Moth_1407  MOTHE_13980/Moth_1408  MOTHE_13990/Moth_1409  MOTHE_14000/Moth_1410  MOTHE_14010/Moth_1411  MOTHE_14020/Moth_1412  MOTHE_14030/Moth_1413  MOTHE_14040/Moth_1414 | ferrous iron transport protein B  ferrous iron transport protein B  ferrous iron transport protein A  electron transport complex subunit RsxB  anaerobic sulfite reductase subunit C  ferredoxin  dinitrogenase iron-molybdenum cofactor  dinitrogenase iron-molybdenum cofactor  hypothetical protein  serine dehydratase alpha chain | **+4.5**  **+4.1**  **+4.4**  **+5.5**  **+5.6**  **+5.6**  **+5.6**  **+5.5**  **+5.8**  **+5.8** | +1.5  +2.2  +2.3  +2.2  +2.1  +2.5  +2.4  +2.5  +3.1  +1.1 | *  *  *  *  +2.1  +3.8  +3.9  +4.0  +4.6  +4.2 |
| MOTHE_18740/Moth_1844  MOTHE_18750/Moth_1845  MOTHE_18760/Moth_1846  MOTHE_18770/Moth_1847  MOTHE_18780/Moth_1848 | **QUINONE BIOSYNTHESIS**  cyclic dehypoxanthine futalosine synthase  chorismate dehydratase  aminodeoxyfutalosine synthase  S-methyl-5'-thioadenosine phosphorylase  fatty acid metabolism regulator protein | **+5.6**  **+5.5**  **+5.4**  **+5.4**  **+5.2** | *  *  *  *  * | -2.4  *  -2.1  -3.6  * |
| MOTHE_09660/Moth_1010  MOTHE_09670/Moth_1011 | Membrane-bound acyltransferase  hypothetical protein (TMH+Signal Peptide) | **-3.9**  **-5.1** | *  +2.1 | *  * |
| MOTHE_04070/Moth_0469  MOTHE_04080/Moth_0470  MOTHE_04090/Moth_0471  MOTHE_04100/none  MOTHE_04110/Moth_0473  MOTHE_04120/Moth_0474 | hypothetical protein  hypothetical protein  Hsp20/alpha crystallin family protein  calcium-transporting ATPase  phosphate-binding protein PstS 1 precursor  heptanoate synthase | *****  *****  *****  *****  **-4.1**  ***** | -2.1  *  *  *  *  * | *  *  *  -2.2  *  +2.0 |
| MOTHE_06660/Moth_0726  MOTHE_06670/Moth_0727  MOTHE_06680/Moth_0728 | hypothetical protein  4-hydroxythreonine-4-phosphate dehydrogenase 2  Gnt-II system L-idonate transporter | **-3.2**  **-3.9**  **-3.3** | *  *  * | *  -2.0  * |
| MOTHE_23570/Moth_2287  MOTHE_23580/Moth_2288  MOTHE_23590/Moth_2289  MOTHE_23600/Moth_2290 | **ARGININE BIOSYNTHESIS**  acetylornithine aminotransferase apoenzyme  N-acetylglutamate kinase  N-acetylglutamate synthase  N-acetyl-gamma-glutamyl-phosphate reductase | *  *  *  * | **+6.9**  **+7.2**  **+7.5**  **+8.0** | *  *  *  * |
| MOTHE_23490/Moth_2279  MOTHE_23500/Moth_2280  MOTHE_23510/Moth_2281  MOTHE_23520/Moth_2282  MOTHE_23530/Moth_2283  MOTHE_23540/Moth_2284  MOTHE_23550/Moth_2285  MOTHE_23560/Moth_2286 | **ARGININE BIOSYNTHESIS**  potassium/proton antiporter  hypothetical protein  Adenine-specific DNA methylase, contains Zn-ribbon  fumarate hydratase subunit beta  fumarase, class I alpha subunit  argininosuccinate lyase  argininosuccinate synthase  ornithine carbamoyltransferase | *  *  *  *  *  *  *  * | **+2.3**  **+2.3**  **+2.1**  **+3.6**  **+3.8**  **+4.4**  **+3.6**  **+3.6** | *  *  *  *  *  *  *  * |
| MOTHE_06370/Moth_0698  MOTHE_06380/Moth_0699  MOTHE_06390/Moth_0700  MOTHE_06400/Moth_0701  MOTHE_06410/Moth_0702 | **ABC TRANSPORTERS**  glucitol operon repressor  D-allose-binding periplasmic protein precursor  ribose import ATP-binding protein RbsA  ribose transport system permease protein RbsC  methylthioribose kinase | *  *  *  * | **+1.7**  **+2.8**  **+4.4**  **+3.7**  **+2.9** | -3.9  -1.8  *  * * |
| MOTHE_22630/Moth_2202  MOTHE_22640/Moth_2203 | **ION TRANSPORT**  hypothetical protein  zinc-transporting ATPase | *  * | **-6.4**  **-4.4** | +2.4  +1.7 |
| MOTHE_04000/Moth_0464  MOTHE_04010/Moth_0465  MOTHE_04020/Moth_0466  MOTHE_04030/Moth_0467 | **ABC TRANSPORTERS**  glycerol dehydrogenase  putative aliphatic sulfonates-binding protein precursor  putative aliphatic sulfonates transport permease SsuC  aliphatic sulfonates import ATP-binding protein SsuB | *  *  *  * | **-5.0**  **-5.2**  **-5.4**  **-4.6** | *  *  *  * |
| MOTHE_20780/Moth_2030  MOTHE_20790/Moth_2031  MOTHE_20800/Moth_2032  MOTHE_20810/Moth_2033  MOTHE_20820/Moth_2034  MOTHE_20830/Moth_2035  MOTHE_20840/Moth_2036  MOTHE_20850/Moth_2037 | **HISTIDINE BIOSYNTHESIS**  phosphoribosyl-ATP pyrophosphatase  imidazole glycerol phosphate synthase subunit HisF  imidazole-4-carboxamide isomerase  imidazole glycerol phosphate synthase subunit HisH 1  imidazoleglycerol-phosphate dehydratase  histidinol dehydrogenase  ATP phosphoribosyltransferase  ATP phosphoribosyltransferase regulatory subunit | *  *  *  *  *  *  *  * | **-2.8**  **-2.6**  **-2.2**  **-3.4**  **-3.8**  **-2.3**  **-1.4**  **-2.6** | *  *  *  *  *  +2.0 +3.0  * |
| MOTHE_03930/Moth_0457  MOTHE_03940/Moth_0458 | **OSMOPROTECTION**  glutamate 2,3-aminomutase  motility protein B | * * | *  * | **+7.7**  **+4.6** |
| MOTHE_19180/Moth_1883  MOTHE_19190/Moth_1884  MOTHE_19200/Moth_1885  MOTHE_19210/Moth_1886  MOTHE_19220/Moth_1887  MOTHE_19230/Moth_1888 | **REDOX**  NADP-reducing hydrogenase subunit HndC  hydrogenase-4 component A  electron transport complex subunit RsxB  NADP-reducing hydrogenase subunit HndA  NADP-reducing hydrogenase subunit HndC  NADPH-Fe(3+) oxidoreductase subunit alpha | *  *  *  *  +2.0  * | *  *  *  *  *  * | **+6.8**  **+4.6**  **+7.6**  **+4.6**  **+4.5**  **+3.7** |
| MOTHE_16910/Moth_1684  MOTHE_16920/Moth_1685  MOTHE_16930/Moth_1686  MOTHE_16940/Moth_1687  MOTHE_16950/Moth_1688  MOTHE_16960/Moth_1689 | **TRANSPORT**  hypothetical protein  glycine betaine/carnitine/choline(GBCC)-binding prot.  GBCC transport system permease protein OpuCD  GBCC transport system permease protein OpuCB  GBCC transport ATP-binding protein OpuCA  DNA-binding transcriptional repressor MngR | *  +2.0  *  *  *  * | *  *  *  *  *  * | **+5.0**  **+5.8**  **+6.0**  **+6.4**  **+5.2**  **+6.4** |
| MOTHE_14320/Moth_1443  MOTHE_14330/Moth_1444  MOTHE_14340/none  MOTHE_14350/none | **TRANSPORT**  demethylmenaquinone methyltransferase  manganese transport system membrane protein MntB  periplasmic solute binding protein family protein  hypothetical protein | *  *  *  * | *  *  *  * | **-6.3**  **-6.2**  **-2.9**  **-6.4** |
| MOTHE_14530/Moth_1464  MOTHE_14540/Moth_1465  MOTHE_14550/Moth_1466  MOTHE_14560/Moth_1467 | **TRANSPORT**  siderophore transport ATP-binding protein YusV  hemin transport system permease protein HmuU  Fe(3+)-citrate-binding protein YfmC precursor  FmdE, molybdenum formylmethanofuran dehydr. | *  *  *  * | *  *  *  * | **-5.6**  **-5.3**  **-5.3**  **-5.8** |
| MOTHE_15200/Moth_1528  MOTHE_15210/Moth_1529  MOTHE_15220/Moth_1530  MOTHE_15230/Moth_1531  MOTHE_15240/Moth_1532  MOTHE_15250/Moth_1533  MOTHE_15260/Moth_1534  MOTHE_15270/Moth_1535  MOTHE_15280/Moth_1536 | **SPORULATION**  phenylalanine-specific permease  stage III sporulation protein AH  hypothetical protein  stage III sporulation protein AF  stage III sporulation protein AE precursor  stage III sporulation protein AC/AD protein stage III sporulation protein AC/AD protein family protein  stage III sporulation protein SpoAB  hypothetical protein | *  *  *  *  *  *  *  *  * | *  *  *  *  *  *  *  *  * | **-2.4**  **-2.8**  **-3.2**  **-3.6**  **-3.4**  **-3.0**  **-3.6**  **-3.8**  **-5.4** |

**Supplementary Table 2. Expression of gene clusters involved in glycolysis in response to cysteine or cadmium.** Numbers indicate FC values from glucose-grown cells in the exponential growth phase with (condition 1: cysteine) or without (control 1) 20 mM L-cysteine or in the early stationary growth phase with (condition 2: cadmium) or without (control 2) 1 mM Cd^2+^ and harvested in the exponential growth phase. *, no significant differential gene. Shown are locus tags of *M. thermoacetica* DSM 521 (MOTHE) and the ATCC39073 (Moth) strain as reference. The numbers (no.) indicate the reactions catalysed in Fig. 4.

| **locus tag** | **predicted function** | **no.** | **cysteine** | **cadmium** | **blue light** |
| --- | --- | --- | --- | --- | --- |
| MOTHE_07780/Moth_0832 | hexokinase | 17 | * | * | * |
| MOTHE_05600/Moth_0610 | phosphoglucoisomerase | 18 | * | * | * |
| MOTHE_05610/Moth_0611 | phosphofructokinase | 19 | +2.6 | * | +2.6 |
| MOTHE_05590/Moth_0609 | fructose bisphosphate aldolase | 20 | * | * | * |
| MOTHE_02790/Moth_0263 | phosphoglycerate kinase | 23 | * | * | * |
| MOTHE_02780/Moth_0262 | glyceraldehyde-3-phosphate dehydrogenase (NAD^+^) | 22 | * | * | * |
| MOTHE_02800/Moth_0264 | triose phosphate isomerase | 21 | * | * | * |
| MOTHE_02810/Moth_0265 | phosphoglycerate mutase | 24 | * | * | * |
| MOTHE_02820/Moth_0266 | enolase | 25 | * | * | * |
| MOTHE_05570/Moth_0607 | pyruvate kinase | 26 | * | * | * |

**Supplementary Table 3. Expression of gene clusters involved in Wood-Ljungdahl in response to cysteine or cadmium.** Numbers indicate FC values from glucose-grown cells in the exponential growth phase with (condition 1: cysteine) or without (control 1) 20 mM L-cysteine or in the early stationary growth phase with (condition 2: cadmium) or without (control 2) 1 mM Cd^2+^ and harvested in the exponential growth phase. *, no significant differential gene. Shown are locus tags of *M. thermoacetica* DSM 521 (MOTHE) and the ATCC39073 (Moth) strain as reference. The numbers (no.) indicate the reactions catalysed in Fig. 4. Genes of potential relevance clustering with described genes are indicated in cursive.

| **locus tag** | **predicted function** | **no.** | **cysteine** | **cadmium** | **blue light** |
| --- | --- | --- | --- | --- | --- |
| MOTHE_23870/Moth_2312  MOTHE_23880/Moth_2313  MOTHE_23890/Moth_2314 | formate dehydrogenase (NADP) alpha subunit  formate dehydrogenase (NADP) alpha subunit  formate dehydrogenase (NADP) beta subunit | 2 | *  *  * | *  *  * | +3.1  +3.2  +3.6 |
| MOTHE_03860/Moth_0450  MOTHE_03870/Moth_0451  MOTHE_03880/Moth_0452 | thiosulfate reductase  Fe-S-cluster-containing dehydrogenase  formate dehydrogenase gamma subunit | 2 | *  *  * | *  *  * | *  *  +2.9 |
| MOTHE_01150/Moth_0109 | Formate-tetrahydrofolate ligase | 3 | +2.1 | * | * |
| MOTHE_15080/Moth_1516 | methenyl-THF cyclohydrolase /5,10-methylene-THF dehydrogenase (NADP^+^) | 4&5 | * | * | * |
| MOTHE_11670/Moth_1191  MOTHE_11680/Moth_1192  MOTHE_11690/Moth_1193  MOTHE_11700/Moth_1194  MOTHE_11710/Moth_1195  MOTHE_11720/Moth_1196 | 5,10-methylenetetrahydrofolate reductase  Methylene-tetrahydrofolate reductase  F420-non-reducing hydrogenase subunit D  NADPH-dependent glutamate synthase beta  heterodisulfide reductase subunit B  heterodisulfide reductase subunit C | 6 | *  *  *  *  *  * | *  *  *  *  *  * | *  *  *  *  *  * |
| MOTHE_11730/Moth_1197 | methyltetrahydrofolate--corrinoid iron-sulfur protein Co-methyltransferase | 7 | * | * | * |
| *MOTHE_11740/Moth_1198*  *MOTHE_11750/Moth_1199*  *MOTHE_11760/Moth_1200*  *MOTHE_11770/Moth_1201*  MOTHE_11780/Moth_1202  MOTHE_11790/Moth_1203  *MOTHE_11800/Moth_1204* | *corrinoid/iron-sulfur protein small subunit*  *septum site-determining protein MinD*  *Na^+^-translocating NADH-quinone reductase F*  *corrinoid/iron-sulfur protein large subunit*  CO dehydrogenase/acetyl-CoA synthase alpha  CO dehydrogenase/acetyl-CoA synthase beta  *septum site-determining protein MinD* | 8 | *  *  *  *  *  *  * | *  *  *  *  *  *  * | *  +2.3  *  *  *  *  * |
| MOTHE_00680/Moth_0064 | pyruvate-ferredoxin/flavodoxin oxidoreductase | 9 | * | * | * |
| MOTHE_03050/Moth_0376  MOTHE_03060/Moth_0377  MOTHE_03070/Moth_0378  MOTHE_03080/Moth_0379 | NADH-dependent phenylglyoxylate DH gamma  pyruvate synthase subunit PorD  pyruvate synthase subunit PorA  NADH-dependent phenylglyoxylate DH beta | 9 | *  +3.0  *  * | +2.8  *  +2.3  * | +2.2  *  *  * |
| MOTHE_15890/Moth_1591  MOTHE_15900/Moth_1592  MOTHE_15910/Moth_1593  MOTHE_15920/none  *MOTHE_15930/Moth_1594* | pyruvate synthase subunit PorB  NADH-dep.phenylglyoxylate DH subunit alpha  pyruvate synthase subunit PorD  hypothetical protein  *ATP-dependent zinc metalloprotease FtsH* | 9 | *  *  *  *  +2.0 |  | *  *  +2.2  +2.1  * |
| MOTHE_19590/Moth_1921  MOTHE_19600/Moth_1922  MOTHE_19610/Moth_1923  MOTHE_19620/Moth_1924 | pyruvate synthase subunit PorB  pyruvate synthase subunit PorA  pyruvate synthase subunit PorD  pyruvate synthase subunit PorC | 9 | *  *  *  * | *  *  *  * | +2.0  *  +3.2  +2.9 |
| MOTHE_11560/Moth_1181 | putative phosphotransacetylase | 10 | * | * | * |
| MOTHE_08100/Moth_0864 | putative phosphotransacetylase | 10 | * | * | * |
| MOTHE_08960/Moth_0940 | acetate kinase | 11 | * | * | -2.6 |

**Supplementary Table 4. Expression of gene clusters involved in energy conservation and redox metabolism in response to cysteine or cadmium.** Numbers indicate FC values from glucose-grown cells in the exponential growth phase with (condition 1: cysteine) or without (control 1) 20 mM L-cysteine or in the early stationary growth phase with (condition 2: cadmium) or without (control 2) 1 mM Cd^2+^ and harvested in the exponential growth phase. *, no significant differential gene. Shown are locus tags of *M. thermoacetica* DSM 521 (MOTHE) and the ATCC39073 (Moth) strain as reference. The numbers (no.) indicate the reactions catalysed in Fig. 4. Genes of potential relevance clustering with described genes are indicated in cursive.

| **locus tag** | **predicted function** | **no.** | **cysteine** | **cadmium** | **blue light** |
| --- | --- | --- | --- | --- | --- |
| MOTHE_19180/Moth_1883  MOTHE_19190/Moth_1884  MOTHE_19200/Moth_1885  MOTHE_19210/Moth_1886  MOTHE_19220/Moth_1887  MOTHE_19230/Moth_1888 | **NADP^+^ reducing hydrogenase**  NADP-reducing hydrogenase subunit HndC  hydrogenase-4 component A  electron transport complex subunit RsxB  NADP-reducing hydrogenase subunit HndA  NADP-reducing hydrogenase subunit HndC  NADPH-Fe^3+^ oxidoreductase subunit alpha | 12a | *  *  *  *  +2.0  * | *  *  *  * *  * | +6.8  +4.6  +7.6  +4.6  +4.5  +3.7 |
| MOTHE_17260/Moth_1717  MOTHE_17270/Moth_1718  MOTHE_17280/Moth_1719 | **bifurcating hydrogenase**  NADP-reducing hydrogenase subunit HndC  NADP-reducing hydrogenase subunit HndC  NADP-reducing hydrogenase subunit HndA | 12b | *  -2.0  -2.2 | +2.2  +2.3  +2.1 | +2.1  +2.2  * |
| MOTHE_15090/Moth_1517  MOTHE_15110/Moth_1518 | **Nfn**  sulfide dehydrogenase (flavoprotein) SudA  sulfide dehydrogenase (flavoprotein) SudB | 13 | *  * | *  * | *  * |
| MOTHE_24560/Moth_2377  MOTHE_24570/Moth_2378  MOTHE_24580/Moth_2379  MOTHE_24590/Moth_2380  MOTHE_24600/Moth_2381  MOTHE_24610/Moth_2382  MOTHE_24620/Moth_2383  MOTHE_24630/Moth_2384 | **F_1_F_O_ ATP synthase**  ATP synthase F1 subcomplex epsilon subunit  ATP synthase F1 subcomplex beta subunit  ATP synthase F1 subcomplex gamma subunit  ATP synthase F1 subcomplex alpha subunit  ATP synthase F1 subcomplex delta subunit  ATP synthase F0 subcomplex B subunit  ATP synthase F0 subcomplex C subunit  ATP synthase F0 subcomplex A subunit | 14 | *  *  *  *  *  *  *  * | *  *  *  *  *  *  *  * | *  *  *  *  *  *  *  -2.0 |
| MOTHE_09340/Moth_0977  MOTHE_09350/Moth_0978  MOTHE_09360/Moth_0979  MOTHE_09370/Moth_0980  MOTHE_09380/Moth_0981  MOTHE_09390/Moth_0982  MOTHE_09400/Moth_0983  MOTHE_09410/Moth_0984  MOTHE_09420/Moth_0985  MOTHE_09430/Moth_0986  MOTHE_09440/Moth_0987 | **Ech1**  NADH dehydrogenase subunit A  NADH dehydrogenase subunit B  NADH dehydrogenase subunit C  NADH dehydrogenase subunit D  NADH dehydrogenase subunit H  NADH dehydrogenase subunit I  NADH dehydrogenase subunit J  NADH dehydrogenase subunit K  NADH dehydrogenase subunit L  NADH dehydrogenase subunit M  NADH dehydrogenase subunit N | 15 | *  *  *  *  *  *  *  *  *  *  * | *  *  *  *  * *  *  *  *  *  * | *  *  +2.5  *  -2.6 *  -2.2  *  *  *  * |
| *MOTHE_22340/Moth_2175*  *MOTHE_22350/Moth_2176*  *MOTHE_22360/Moth_2177*  *MOTHE_22370/Moth_2178*  *MOTHE_22380/Moth_2179*  *MOTHE_22390/Moth_2180*  *MOTHE_22400/Moth_2181*  *MOTHE_22410/Moth_2182*  *MOTHE_22420/Moth_2183*  MOTHE_22430/Moth_2184  MOTHE_22440/Moth_2185  MOTHE_22450/Moth_2186  MOTHE_22460/Moth_2187  MOTHE_22470/Moth_2188  MOTHE_22480/Moth_2189  MOTHE_22490/Moth_2190  MOTHE_22500/Moth_2191  MOTHE_22510/Moth_2192  MOTHE_22520/Moth_2193  MOTHE_22530/Moth_2194 | **Ech2 (Fhl)**  *FdhD protein*  *Hydrogenase maturation, HypE*  *Hydrogenase maturation protein HypD*  *hydrogenase expression/formation HypC*  *hydrogenase maturation protein HypF*  *hydrogenase nickel incorporation protein HypB*  *Hydrogenase-3 nickel incorporation HypA*  *Hydrogenase 3 maturation peptidase Hycl*  *formate hydrogenlyase maturation HycH*  hypothetical protein  NADH dehydrogenase subunit I  hypothetical protein  hydrogenase-4 component D  hydrogenase-4 component F  hydrogenase-4 component E  hydrogenase-4 component C  hydrogenase-4 component B  hydrogenase-4 component A  formate dehydrogenase major subunit  formate/nitrite transporter | 16 | *  *  *  *  *  *  *  *  *  *  *  *  *  *  *  *  *  *  *  * | *  *  *  *  *  *  *  *  *  *  *  *  *  *  *  *  *  *  *  * | *  *  *  *  -2.5  *  *  -2.6  *  *  -3.1  -3.4  *  -2.3  *  *  *  *  *  * |

**Supplementary Table 5. Expression of gene clusters involved in cysteine and sulfur metabolism in response to cysteine or cadmium.** Numbers indicate FC values from glucose-grown cells in the exponential growth phase with (condition 1: cysteine) or without (control 1) 20 mM L-cysteine or in the early stationary growth phase with (condition 2: cadmium) or without (control 2) 1 mM Cd^2+^ and harvested in the exponential growth phase. *, no significant differential gene. Shown are locus tags of *M. thermoacetica* DSM 521 (MOTHE) and the ATCC39073 (Moth) strain as reference.

| **locus tag** | **predicted function** | **E.C.** | **cysteine** | **cadmium** | **blue light** |
| --- | --- | --- | --- | --- | --- |
| MOTHE_18540/Moth_1826 | malate dehydrogenase (NAD) | 1.1.1.27 | * | * | +2.1 |
| MOTHE_12910/Moth_1307 | homoserine dehydrogenase | 1.1.1.3 | * | * | * |
| MOTHE_00190/Moth_0020 | D-3-phosphoglycerate dehydrogenase | 1.1.1.95 | * | * | * |
| MOTHE_10260/Moth_1066 | aspartate semialdehyde dehydrogenase | 1.2.1.11 | * | * | * |
| MOTHE_11380/Moth_1168 | anaerobic sulfite reductase subunit C | 1.8.1.- | * | * | * |
| MOTHE_11390/Moth_1169 | anaerobic sulfite reductase subunit B | 1.8.1.- | * | * | * |
| MOTHE_11400/Moth_1170 | anaerobic sulfite reductase subunit A | 1.8.1.- | * | * | * |
| MOTHE_00230/Moth_0024 | thiosulfate dehydrogenase [quinone] large subunit | 1.8.5.2 | * | * | * |
| MOTHE_13690/Moth_1384 | anaerobic dimethyl sulfoxide reductase subunit C | 1.8.5.3 | * | * | * |
| MOTHE_13700/Moth_1385 | anaerobic dimethyl sulfoxide reductase subunit B | 1.8.5.3 | * | * | * |
| MOTHE_13710/Moth_1386 | anaerobic dimethyl sulfoxide reductase subunit A | 1.8.5.3 | * | * | * |
| MOTHE_13720/Moth_1388 | anaerobic dimethyl sulfoxide reductase subunit B | 1.8.5.3 | * | * | * |
| MOTHE_03860/Moth_0450 | thiosulfate reductase / polysulfide reductase chain A | 1.8.5.5 | * | * | * |
| MOTHE_15990/Moth_1600 | dissimilatory sulfite reductase beta subunit | 1.8.99.5 | * | * | * |
| MOTHE_16000/Moth_1601 | dissimilatory sulfite reductase alpha subunit | 1.8.99.5 | * | * | * |
| MOTHE_16350/Moth_1629 | dissimilatory sulfite reductase beta subunit | 1.8.99.5 | * | * | * |
| MOTHE_16360/Moth_1630 | dissimilatory sulfite reductase alpha subunit | 1.8.99.5 | * | * | * |
| MOTHE_13030/Moth_0385 | 5-methyltetrahydrofolate--homocysteine methyltransferase | 2.1.1.13 | * | * | * |
| MOTHE_03190/Moth_0387 | 5-methyltetrahydrofolate--homocysteine methyltransferase | 2.1.1.13 | * | * | * |
| MOTHE_11840/Moth_1207 | 5-methyltetrahydrofolate--homocysteine methyltransferase | 2.1.1.13 | * | * | * |
| MOTHE_11850/Moth_1208 | 5-methyltetrahydrofolate--homocysteine methyltransferase | 2.1.1.13 | * | +2.1 | * |
| MOTHE_13000/Moth_1316 | 5-methyltetrahydrofolate--homocysteine methyltransferase | 2.1.1.13 | * | * | * |
| MOTHE_03140/Moth_1318 | 5-methyltetrahydrofolate--homocysteine methyltransferase | 2.1.1.13 | * | * | * |
| MOTHE_21690/Moth_2115 | 5-methyltetrahydrofolate--homocysteine methyltransferase | 2.1.1.13 | * | * | * |
| MOTHE_25700/Moth_2485 | serine O-acetyltransferase | 2.3.1.30 | * | * | * |
| MOTHE_12920/Moth_1308 | homoserine O-acetyltransferase / homoserine O-succinyltransferase | 2.3.1.46 | * | * | * |
| MOTHE_06440/Moth_0705 | methylthioadenosine phosphorylase | 2.4.2.28 | * | * | +2.6 |
| MOTHE_18410/Moth_1816 | spermidine synthase | 2.5.1.16 | * | * | * |
| MOTHE_17130/Moth_1706 | cysteine synthase A (cysK1) | 2.5.1.47 | * | * | * |
| MOTHE_20120/Moth_1971 | cysteine synthase / O-acetylserine sulfhydrylase (cysK2) | 2.5.1.47 | * | -2.4 | * |
| MOTHE_20430/Moth_2000 | cysteine synthase (cysK4) | 2.5.1.47 | +4.4 | * | * |
| MOTHE_08430/Moth_0894 | methionine adenosyltransferase | 2.5.1.6 | * | * | * |
| MOTHE_14800/Moth_1490 | L-aspartate aminotransferase apoenzyme | 2.6.1.1 | * | * | * |
| MOTHE_23270/Moth_2260 | branched chain amino acid aminotransferase apoenzyme | 2.6.1.42 | * | * | * |
| MOTHE_10270/Moth_1067 | aspartate kinase | 2.7.2.4 | * | * | * |
| MOTHE_12880/Moth_1304 | aspartate kinase | 2.7.2.4 | * | * | -2.3 |
| MOTHE_03690/Moth_0433 | putative sulfurtransferase DsrE | 2.8.1.- | * | * | * |
| MOTHE_03720/Moth_0436 | sulfurtransferase TusA | 2.8.1.- | * | * | * |
| MOTHE_16320/Moth_1626 | tRNA 2-thiouridine synthesizing protein A / sulfurtransferase TusA | 2.8.1.- | * | * | * |
| MOTHE_16370/Moth_1631 | sulfurtransferase TusE | 2.8.1.- | * | * | +2.5 |
| MOTHE_16700/Moth_1663 | thiazole synthase | 2.8.1.10 | * | * | * |
| MOTHE_16560/Moth_1650 | tRNA-specific 2-thiouridylase MnmA | 2.8.1.13 | * | * | * |
| MOTHE_02090/Moth_0198 | putative tRNA sulfurtransferase | 2.8.1.4 | * | * | * |
| MOTHE_03820/Moth_0446 | biotin synthase | 2.8.1.6 | * | * | * |
| MOTHE_17460/Moth_1735 | biotin synthase | 2.8.1.6 | +3.1 | * | * |
| MOTHE_02100/Moth_0199 | cysteine desulfurase IscS | 2.8.1.7 | * | * | * |
| MOTHE_13930/Moth_1403 | putative cysteine desulfurase | 2.8.1.7 | * | * | * |
| MOTHE_16590/Moth_1652 | cysteine desulfurase IscS | 2.8.1.7 | * | * | * |
| MOTHE_22760/Moth_2214 | cysteine desulfurase (sufS) | 2.8.1.7/4.4.1.16 | * | * | * |
| MOTHE_17760/Moth_1762 | lipoyl synthase | 2.8.1.8 | * | * | -2.0 |
| MOTHE_10110/Moth_1052 | phosphoesterase RecJ domain-containing protein | 3.1.3.7/3.1.13.3 | * | * | * |
| MOTHE_06450/Moth_0706 | adenosylhomocysteinase | 3.3.1.1 | * | * | * |
| MOTHE_09200/Moth_0963 | 5-methylthioadenosine/S-adenosylhomocysteine deaminase | 3.5.4.31/28 | * | * | * |
| MOTHE_12630/Moth_1282 | adenosylmethionine decarboxylase proenzyme | 4.1.1.50 | * | * | * |
| MOTHE_19850/Moth_1946 | L-serine ammonia-lyase | 4.3.1.17 | +3.7 | * | * |
| MOTHE_19860/Moth_1947 | L-serine ammonia-lyase | 4.3.1.17 | +3.6 | * | * |
| MOTHE_20310/Moth_1989 | cysteine synthase /cystathionine gamma-synthase | 4.4.1.1/2 | * | * | * |
| MOTHE_10800/Moth_1119 | methionine gamma-lyase | 4.4.1.11 | * | * | * |
| MOTHE_12930/Moth_1309 | O-acetylhomoserine sulfhydrylase / methionine gamma-lyase | 4.4.1.11 | * | * | * |
| MOTHE_20320/Moth_1990 | cystathionine beta-lyase | 4.4.1.13/8 | * | * | * |
| MOTHE_01240/Moth_0118 | phosphosulfolactate synthase | 4.4.1.19 | * | * | * |
| MOTHE_19650/none | (2R)-sulfolactate sulfo-lyase subunit beta | 4.4.1.24 | * | * | * |
| MOTHE_14040/Moth_1414 | L-cysteine desulfidase | 4.4.1.28 | +5.8 | * | +4.2 |
| MOTHE_06420/Moth_0703 | methylthioribose-1-phosphate isomerase | 5.3.1.23 | * | * | * |

**Supplementary Table 6. Basic data of transcriptome.**

| **Condition** | **Sample** | **Total reads** | **Trimmed reads** | **Mapped reads** | **Mapping rate [%]** |
| --- | --- | --- | --- | --- | --- |
| cysteine | MOAT4 | 19.802.886 | 19.789.738 | 12.006.996 | 60,67280 |
| cysteine | MOAT5 | 18.784.709 | 18.773.471 | 11.703.679 | 62,34160 |
| cysteine | MOAT6 | 17.312.086 | 17.320.207 | 11.816.399 | 68,29420 |
| glucose exp | MOAT7 | 19.715.533 | 19.704.212 | 12.614.470 | 64,01920 |
| glucose exp | MOAT8 | 16.351.837 | 16.342.137 | 10.751.947 | 65,79280 |
| glucose exp | MOAT9 | 17.254.605 | 17.244.235 | 11.112.511 | 64,44190 |
| glucose stat | MOAT16 | 28.094.780 | 28.065.315 | 16.050.760 | 57,19070 |
| glucose stat | MOAT17 | 47.316.675 | 47.242.717 | 23.852.091 | 50,48840 |
| glucose stat | MOAT18 | 59.997.655 | 59.919.939 | 14.746.940 | 24,61110 |
| cadmium | MOAT19 | 24.056.208 | 24.035.230 | 6.940.253 | 28,87530 |
| cadmium | MOAT20 | 36.815.291 | 36.777.181 | 16.006.966 | 43,52420 |
| cadmium | MOAT21 | 30.412.270 | 30.384.274 | 9.057.048 | 29,80830 |
| blue light | MOAT22 | 26.490.277 | 26.465.829 | 7.316.906 | 27,64660 |
| blue light | MOAT23 | 108.203.270 | 108.128.460 | 8.302.007 | 7,67791 |
| blue light | MOAT24 | 114.489.763 | 114.407.999 | 6.375.744 | 5,57281 |

**Supplementary Text 1**

The *MOTHE_04070-04120* cluster encoding the Hsp also comprises two hypothetical proteins, a P-type ATPase, a phosphate ABC transporter substrate binding protein and a fructose-bisphosphate aldolase (FBA). *MOTHE_04120* is a second FBA found in the genome and belongs to the class Ia, DhnA family (COG1830, pfam01791), whereas the isogene (*MOTHE_05590*) is embedded in context of other glycolytic genes (which are also upregulated under cysteine abundance; see below) encodes a class I aldolase (pfam01116). The aldolase catalyzes the cleavage reaction of fructose-1,6-bisphosphate to glyceraldehyde-3-phosphate (GAP) and dihydroxyacetone phosphate (DHAP). Aldolase have been shown to bind V-ATPases in kidney epithelial cells and yeast, regulating their assembly, protein production and activity ^4,5^. This could also be true for the aldolase and P-type ATPase in *M. thermoacetica*.

The *MOTHE_06660-06680* genes encode a D-threonate/D-erythronate kinase (DUF1537), a 4-hydroxythreonine-4-phosphate dehydrogenase (PdxA2) and a gluconate:H^+^ symporter (GntP family). Homologues of these genes from *Cupriavidus necator* have been shown to catalyze the binding, transport and conversion of the sugar acid D-threonate (which is a degradation product of ascorbate) to DHAP and CO_2_ ^6^. Both clusters putatively involved in transport and metabolism (*MOTHE_06660-06680* and *MOTHE_09660-9670*) were also downregulated in cells exposed to Cd, suggesting this may also be a general response to stress rather than to cysteine alone.

**Supplementary Text 2**

The FDH which catalyzes the first step of the WLP (Fig. 4, step 2), the reduction of CO_2_ to formate with NADH, has been purified and biochemically characterized ^7^. The measured formate:NADP^+^ oxidoreductase activities in CEs of normal or CdS cells were in the same range and lower in the stationary phase. The FDH catalyzing this activity is encoded by the *MOTHE_23870-23890* cluster, which was only slightly upregulated in response to cadmium (below the FC threshold with an FC_cadmium_=+1.7), but not cysteine. The similar specific activities despite a slightly higher expression with cadmium indicates that Cd^2+^ could prevent proper maturation or inhibit the enzyme activity. Besides the NADP-dependent FDH, there are two additional clusters which encode formate dehydrogenases: the *MOTHE_22520* encoded just downstream of the *ech2* genes and the *MOTHE_03860-03880* genes. The latter encodes the thiosulfate reductase/formate dehydrogenase, which contains a molybdopterin-binding domain and a signal peptide, a 4Fe-4S ferredoxin and a formate dehydrogenase gamma subunit, which is membrane-bound (four transmembrane helices [TMH]) and possesses a di-haem cytochrome domain. The three proteins are reminiscent of the formate dehydrogenase-N (Fdn-N) from *E. coli*, whose menaquinone reduction site is associated with a possible proton pathway contributing to the electrochemical proton gradient by a redox loop ^8^. This enzyme has not been biochemically characterized in *M. thermoacetica* yet. The Ech2-associated FDH gene was slightly downregulated with cadmium but not cysteine (below the FC threshold with an FC_cadmium_=-1.6). In *E. coli*, this FDH is part of the FHL complex which links the interconversion of formate to H_2_ and CO_2_ to the chemiosmotic gradient ^9^. This function may also be true for the *M. thermoacetica* homologue, but the FDH gene is not on the same transcript and expression of the genes is quite different. The gene encoding the formate-THF ligase (Fig. 4, step 3) was upregulated with cysteine (FC=+2.1), the methenyl-THF cyclohydrolase and dehydrogenase (Moth_1516) was not significantly effected (step 4 and 5) and the methylene-THF reductase (MTHFR; step 6), the methyltransferase (step 7) and CODH genes (step 8) were not DEGs either. The MTHFR of *M. thermocetica* is a particularly interesting enzyme, as there is evidence that it is a hexaheteromeric complex which couples NADH oxidation to the exergonic reduction of methylene-THF and the simultaneous reduction of a yet unidentified second electron acceptor via flavin-based electron bifurcation (FBEB) ^10^. The acetyl-CoA produced from the CODH/Acs is then further converted to pyruvate by a PFOR (Fig. 4, step 9). Such an enzyme has been purified and biochemically characterized from this organism ^11^, but there were no statistically relevant changes in expression of the respective *MOTHE_00680* gene. *M. thermoacetica* possesses two phosphotransacetylase (PTA)-encoding genes catalyzing the conversion of acetyl-CoA to acetyl-phosphate (Fig. 4, step 10), but neither the other *pta* gene (*MOTHE_08100*) nor the acetate kinase, catalyzing the last step (step 11) of converting acetyl-phosphate to acetate under ATP generation from SLP, were significantly differentially expressed.

**Supplementary Text 3**

The HydABC has been purified and characterized and its function is to produce H_2_ when cells grow on sugars ^12^. The NADP^+^ reducing hydrogenase is not so well characterized yet, but it has been reported that activity is present in H_2_+CO_2_-grown cells but hardly undetectable in glucose-grown cells ^13^. The cluster comprises six genes which encode a ferredoxin hydrogenase, an electron transport protein HydN, a 4Fe-4S binding domain-containing protein, an NADH dehydrogenase subunit E, an NADH-quinone oxidoreductase subunit F and a 4Fe-4S dicluster domain-containing protein. Homologues of these proteins in *Clostridium autoethanogenum* form a complex with a formate dehydrogenase which catalyzes the reversible reduction of Fd and NADP^+^ with H_2_ or formate and the reversible formation of H_2_ and CO_2_ from formate ^14^. The NADP^+^ reducing hydrogenase could form a comparable complex in *M. thermoacetica*; however, the membrane-bound and cytochrome-associated FDH complex (*MOTHE_03860*) was not a DEG with cysteine (FC=+1.2).

**Supplementary Text 4**

The genes in context of the Csd encode to MinD superfamily P-loop ATPase (with a ferredoxin domain) proteins, a 4Fe-4S dicluster domain-containing protein, two predicted Fe-Mo cluster-binding proteins (NifX family) and a hypothetical protein. The Fe-Mo cluster binding proteins are involved in the biosynthesis of the cofactor for the nitrogenase (*MOTHE_04980*) for example, and the ATPase proteins possess domains found in cobyrinic acid a,c-diamide synthetase which catalyze the first step in biosynthesis of cobalamin (vitamin B_12_). Inspection of the Csd sequence showed that it belongs to the same two protein families as the enzyme of *Methanocaldococcus jannaschii*, the serine dehydratase-like, alpha subunit (IPR005130; Pfam PF03313), and an uncharacterised protein family UPF0597 (IPR021144). The genetic context is however different, since *MJ1025* is only accompanied by one other gene (*MJ1026*) encoding the thiamine biosynthesis protein ThiC. Nevertheless, the *M. jannaschii* enzyme could be stimulated by MV by 40% ^15^, supporting the notion that a yet unknown cofactor may be involved in the catalyses of the Csd enzyme in *M. thermoacetica*. Insufficient amounts of this putative cofactor in the CE may have been the reason that cysteine desulfhydrase activity was not detectable in CEs with the method used in this work.

**References**

1 Sakimoto, K. K., Wong, A. B. & Yang, P. Self-photosensitization of nonphotosynthetic bacteria for solar-to-chemical production. *Science* **351**, 74-77, (2016).

2 Müller, V. Microbes in a knight's armor. *Science* **351**, 34, (2016).

3 Luo, W. & Brouwer, C. Pathview: an R/Bioconductor package for pathway-based data integration and visualization. *Bioinformatics* **29**, 1830-1831, (2013).

4 Nakamura, S. Glucose activates H^+^-ATPase in kidney epithelial cells. *American journal of physiology. Cell physiology* **287**, C97-105, (2004).

5 Lu, M., Sautin, Y. Y., Holliday, L. S. & Gluck, S. L. The glycolytic enzyme aldolase mediates assembly, expression, and activity of vacuolar H^+^-ATPase. *J Biol Chem* **279**, 8732-8739, (2004).

6 Zhang, X. *et al.* Assignment of function to a domain of unknown function: DUF1537 is a new kinase family in catabolic pathways for acid sugars. *Proc Natl Acad Sci U S A* **113**, E4161-4169, (2016).

7 Li, L. F., Ljungdahl, L. & Wood, H. G. Properties of nicotinamide adenine dinucleotide phosphate-dependent formate dehydrogenase from *Clostridium thermoaceticum*. *J Bacteriol* **92**, 405-412, (1966).

8 Jormakka, M., Tornroth, S., Byrne, B. & Iwata, S. Molecular basis of proton motive force generation: structure of formate dehydrogenase-N. *Science* **295**, 1863-1868, (2002).

9 McDowall, J. S. *et al.* Bacterial formate hydrogenlyase complex. *Proc Natl Acad Sci U S A* **111**, E3948-E3956, (2014).

10 Mock, J., Wang, S., Huang, H., Kahnt, J. & Thauer, R. K. Evidence for a hexaheteromeric methylenetetrahydrofolate reductase in *Moorella thermoacetica*. *J Bacteriol* **196**, 3303-3314, (2014).

11 Furdui, C. & Ragsdale, S. W. The roles of coenzyme A in the pyruvate:ferredoxin oxidoreductase reaction mechanism: rate enhancement of electron transfer from a radical intermediate to an iron-sulfur cluster. *Biochemistry* **41**, 9921-9937, (2002).

12 Wang, S., Huang, H., Kahnt, J. & Thauer, R. K. A reversible electron-bifurcating ferredoxin- and NAD-dependent [FeFe]-hydrogenase (HydABC) in *Moorella thermoacetica*. *J Bacteriol* **195**, 1267-1275, (2013).

13 Huang, H., Wang, S., Moll, J. & Thauer, R. K. Electron bifurcation involved in the energy metabolism of the acetogenic bacterium *Moorella thermoacetica* growing on glucose or H_2_ plus CO_2_. *J Bacteriol* **194**, 3689-3699, (2012).

14 Wang, S. *et al.* NADP-specific electron-bifurcating [FeFe]-hydrogenase in a functional complex with formate dehydrogenase in *Clostridium autoethanogenum* grown on CO. *J Bacteriol* **195**, 4373-4386, (2013).

15 Tchong, S. I., Xu, H. & White, R. H. L-cysteine desulfidase: an [4Fe-4S] enzyme isolated from *Methanocaldococcus jannaschii* that catalyzes the breakdown of L-cysteine into pyruvate, ammonia, and sulfide. *Biochemistry* **44**, 1659-1670, (2005).
